# The MarR family regulator RmaH underlies the trade-off between acid sensitivity and antibiotic tolerance in *Lactococcus lactis*

**DOI:** 10.1128/jb.00502-25

**Published:** 2026-05-06

**Authors:** Qianqian Song, Peng Zhang, Hao Wu, Hongji Zhu, Jiaheng Liu, Jianjun Qiao

**Affiliations:** 1 Department of Pharmaceutical and Biological Engineering, School of Chemical Engineering, Sichuan University 12530, Chengdu, China; 2 State Key Laboratory of Synthetic Biology, Key Laboratory of Systems Bioengineering (Ministry of Education), School of Synthetic Biology and Biomanufacturing, Tianjin University 12605, Tianjin, China; 3 Zhejiang Institute of Tianjin University (Shaoxing) 12605, Shaoxing, China; The Ohio State University, Columbus, Ohio, USA

**Keywords:** *Lactococcus lactis*, transcription regulator, RmaH, acid sensitivity, antibiotic tolerance

## Abstract

Microorganisms exhibit remarkable adaptability to diverse environmental stresses through transcriptional reprogramming. Here, we focused on the MarR family regulator RmaH in *Lactococcus lactis*, which orchestrated a pivotal trade-off: enhancing tolerance to the bacteriocin nisin and the antibiotic vancomycin at the expense of acid resistance. RmaH directly activated genes for cell wall and membrane lipid biosynthesis, leading to a thicker cell wall, and repressed the arginine deiminase pathway, attenuating acid tolerance. Integrated transcriptomic, chromatin immunoprecipitation-sequencing, and electrophoretic mobility shift assay identified RmaH as a global transcription regulator, modulating processes, including amino acid transport and metabolism, cell wall/membrane/envelope biogenesis, carbohydrate transport and metabolism, and nucleotide transport and metabolism. The molecular basis of this regulation was defined by identifying two RmaH-specific DNA-binding motifs and confirming the essential role of arginine residues R79 and R87 in DNA binding. Our work elucidated a sophisticated regulatory mechanism that enabled bacteria to navigate complex and changing stressors.

**IMPORTANCE:**

This study reveals how the global regulator RmaH in *Lactococcus lactis* orchestrates a critical survival trade-off, prioritizing antibiotic tolerance over acid resistance. By directly activating cell wall and membrane biosynthesis pathways, RmaH enhances defense against antimicrobials like nisin and vancomycin. Concurrently, it represses the arginine deiminase pathway, compromising the cell’s ability to mitigate acid stress. This work provides a fundamental model for how bacteria dynamically allocate finite cellular resources to navigate complex, changing environments. The elucidated mechanism offers broader insights into bacterial persistence strategies and the physiological compromises underlying stress response networks.

## INTRODUCTION

The survival and proliferation of bacteria depend on their rapid response and adaptation to fluctuating environments, including temperature change, pH shift, nutritional availability, antibiotics exposure, competition from symbiotic microorganisms, etc. (1–3). Various signal-sensing and transduction mechanisms are utilized by bacteria to modulate gene expression, thereby facilitating physiological adaptation. Among these mechanisms, transcription regulatory proteins directly mediate gene transcription via binding to specific DNA sequences, playing pivotal roles in regulating gene expression (4).

The MarR (multiple antibiotic resistance regulator) family of transcriptional regulators represents an ancient regulator family that is widely distributed among archaea and bacteria, predating their evolutionary divergence (5). It was first described in *Escherichia coli* by Cohen et al. (6) that *E. coli* carrying the *marR* mutation displayed multiple antibiotic resistance. Recently, MarR family regulators have been found to be involved in various crucial biological processes (7), including antibiotic resistance, oxidative stress, virulence, and the catabolism of aromatic compounds. These regulators typically possess a winged helix-turn-helix (wHTH) motif and function as dimers by binding to AT-rich palindromic DNA sequences, such as “cATTGAnnnnnTCAATg” recognized by *Rhodococcus jostii* RHA1 CouR (8) and “GTTATAnnnTATAAC” recognized by *Rhodopseudomonas palustris* CouR (9). The conserved arginine residues within the wHTH motif of MarR family proteins are critical for target DNA binding, although the specific interactions between the Arg residue and DNA bases may be different. In *Staphylococcus aureus* MepR, Asp85 and Arg87 interact with the adenine and thymine bases of the DNA minor groove via hydrogen bond interaction (10), while Asp84 and Arg86 in *Salmonella typhimurium* SlyA have been confirmed to contact the DNA minor groove through van der Waals interaction (11).

Although initially considered classical transcriptional repressors regulating local or specific gene expression, recent studies have revealed that several MarR family regulators can also function as global transcription factors, positively and negatively modulating gene expression (7). The MarR family regulator SlyA, highly conserved in *Enterobacteriaceae*, is a global regulator with transcriptional anti-silence function. The SlyA protein can alleviate gene silencing caused by the silencer H-NS by displacing H-NS or binding to the H-NS-DNA complex to remodel its structure (12, 13). SlyA is not unique; the MarR family regulator ScoC is also a global regulator in *Bacillus subtilis* and *Bacillus pumilus*. In *B. subtilis*, ScoC positively and negatively regulates more than 560 genes involved in sporulation, motility, transport, and metabolism of amino acids, carbohydrates, and nucleotides, as well as transcription regulation (14). Similarly, the knockout of ScoC in *B. pumilus* results in 531 upregulated and 467 downregulated genes, and ScoC is identified as a positive regulator of flagella formation and motility (15). Although the mechanism behind the pleiotropy of ScoC remains unclear, it nevertheless provides a typical example of MarR family regulators functioning as global regulators and anti-silencers.

*Lactococcus lactis*, a type of lactic acid bacterium (LAB) widely used in the food fermentation industry, possesses excellent biosafety and has promising applications in biopharmaceuticals, vaccine development, and as intestinal probiotics (16–19). During industrial applications, *L. lactis* encounters various physiological or non-physiological stressors, such as acid, antibiotics, and bile salts. Transcriptional regulatory proteins, i.e., YthA (20), CodY (1), ComX (21), and LssR (22), play considerable roles in regulating the response of *L. lactis* to environmental stresses by modulating gene expression specifically or globally. There are 10 MarR family regulators in *L. lactis* NZ9000, one of which is the zinc-responsive repressor ZitR. ZitR has been found to modulate the response of *L. lactis* MG1363 to Zn^2+^ starvation and promote Zn^2+^ uptake only at extremely low concentrations (23–25). At the molecular level, ZitR can bind to an imperfect palindrome sequence “TTAACYRGTTAA” in the promoter sequence of *zitRSPQ*, encoding ABC uptake systems specific for Zn^2+^, thereby inhibiting its transcription (23). This binding can be inhibited under the induction of very low Zn^2+^ concentration due to the high affinity of ZitR for Zn^2+^ (24, 25). Recently, we also identified RmaH as a MarR-family activator of the *murT-gatD* operon, a regulation that paradoxically reduces acid tolerance in *L. lactis* by altering peptidoglycan charge (26). This finding suggested that RmaH plays a central role in managing cell envelope properties in response to stress. As a result, we postulated that RmaH may also be a crucial regulator of antibiotic susceptibility targeting the cell envelope, possibly controlling a key trade-off in bacterial fitness strategies.

In this study, the influence of the MarR family regulator RmaH on the stress response of *L. lactis* was further studied by the acid, nisin, and vancomycin stress tests. The overexpression of RmaH not only decreased the acid tolerance of *L. lactis* NZ9000 but also contributed to enhanced nisin and vancomycin resistance. Comparative transcriptome analysis, coupled with chromatin immunoprecipitation-sequencing (ChIP-seq) analysis, electrophoretic mobility shift assay (EMSA), stress test, and cell morphology detection, was applied to explore the regulatory mechanism. Furthermore, the DNA-binding motif and key amino acids of the transcriptional regulator RmaH, which are essential for its interactions with target genes, were studied and further verified through EMSA experiments. This study enhanced the understanding of how *L. lactis* strains adapted to antibiotic resistance and acid stress, as well as the balance between the two. The interaction mechanism between RmaH and its target genes elucidated in this study allowed for the identification of other potential cellular processes in which RmaH participates.

## RESULTS

### RmaH is involved in the tolerance of *L. lactis* to nisin, vancomycin, and acid stress

**Fig 1:**
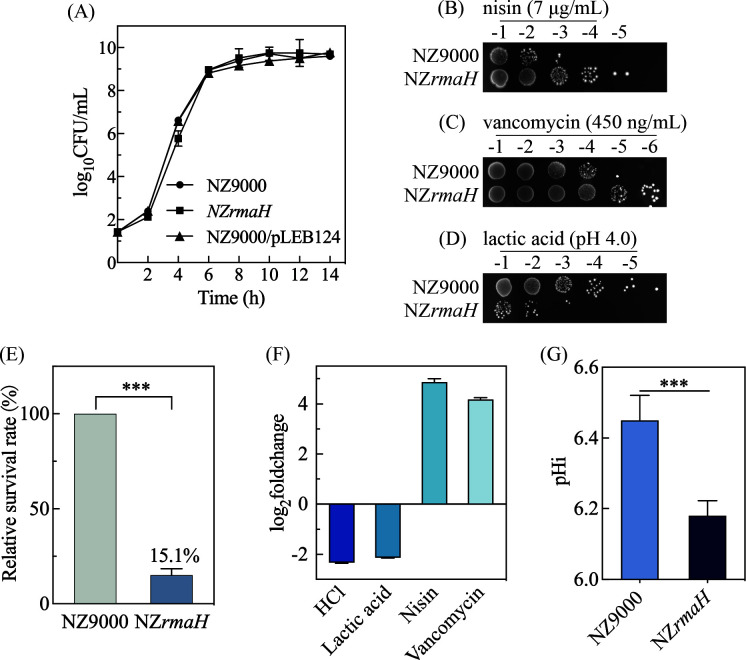
Effect of transcription factor RmaH on the growth and response of *L. lactis* to environmental stress. (**A**) The growth curves of *L. lactis* NZ9000, NZ9000/pLEB124, and NZ*rmaH*. (**B**) Nisin (7 μg/mL) tolerance test, (**C**) vancomycin (450 ng/mL) tolerance test, (**D**) lactic acid (pH 4.0, 3 h) stress test, and (**E**) HCl (pH 3.0, 3 h) stress test (***P < 0.001). (**F**) Changes in *rmaH* transcription levels under different stress conditions. (**G**) Effect of RmaH on intracellular pH (pHi).

The MarR family regulator RmaH has been identified as an activator of the *murT-gatD* operon, which alters peptidoglycan charge, paradoxically reducing the lactic acid tolerance of *L. lactis* NZ9000 (26). The function of RmaH in regulating peptidoglycan prompted us to further investigate its role in resistance to antibiotics targeting the cell envelope. The function of RmaH was identified by gene overexpression and disruption in *L. lactis*. However, several attempts to construct the *rmaH* mutant failed, possibly because *rmaH* is essential. A growth analysis by determining colony-forming unit (CFU) showed that the *rmaH*-overexpressing strain NZ*rmaH* exhibited a slight growth delay in the lag and logarithmic phases but ultimately reached a biomass level similar to NZ9000 (maximum biomass of approximately 1.8 × 10^10^ CFU/mL) (Fig. 1A), which might be because the overexpression of *rmaH* caused a growth burden on NZ9000.

Subsequent investigations focused on RmaH’s influence on the response of *L. lactis* to antibacterial peptide nisin (targeting the peptidoglycan Lipid II), antibiotics vancomycin (targeting D-Ala-D-Ala at the end of Lipid II), and acid stress. As suggested in Fig. S1, the introduction of empty plasmid pLEB124 has no significant effect on the tolerance of *L. lactis* to nisin, vancomycin, and acid stress. Compared with NZ9000, the strain NZ*rmaH* displayed increased tolerance to nisin and vancomycin, and the maximum valid dilutions both increased 10^2^-fold (Fig. 1B and C). In contrast, the NZ*rmaH* exhibited heightened sensitivity to the acid stress (Fig. 1D and E). Exposure to lactic acid stress (pH 4.0) for 3 h resulted in approximately a 10^3^-fold decrease in survival compared to NZ9000, consistent with the recent results (26). Similarly, the cell viability of NZ*rmaH* was just 15.1% ± 2.4% of that of NZ9000 under the hydrochloric acid (HCl) stress (pH 3.0, 3 h).

Furthermore, the *rmaH* transcription level changes in NZ9000 were assessed after exposure to stress condition for 3 h: HCl (pH 3.0), lactic acid (pH 4.0), nisin (7 μg/mL), and vancomycin (450 ng/mL), relative to growth in normal medium (Fig. 1F). The transcription levels of *rmaH* were downregulated (−2.34 and −2.13-fold, Log_2_foldchange [stress condition/normal medium]) in response to HCl and lactic acid stress, indicating its involvement in acid tolerance mechanisms. Conversely, the transcription of *rmaH* was significantly upregulated (4.86- and 4.22-fold) under nisin and vancomycin stress conditions, suggesting a role of RmaH in antibacterial peptide and antibiotic response pathways.

### RmaH positively regulates cell wall thickness

**Fig 2:**
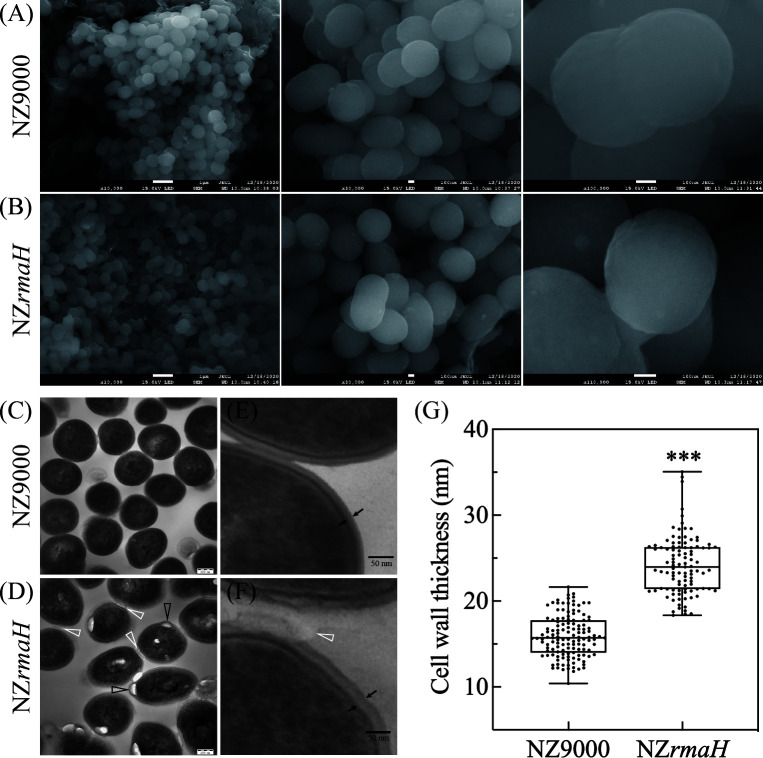
Effect of RmaH on cell morphology of *L. lactis*. SEM observation of strains (**A**) NZ9000 and (**B**) NZ*rmaH*; TEM observation of strains NZ9000 (**C and E**) and NZ*rmaH* (**D and F**); (**G**) statistical analysis of cell wall thickness (***P < 0.001). The range indicated by the two black arrows in panels **E and F** shows the measurement range of the cell wall.

The fact that RmaH enhanced the tolerance of *L. lactis* to nisin and vancomycin targeted at the cell wall peptidoglycan Lipid II prompted us to hypothesize that overexpression of *rmaH* might affect the biosynthesis of peptidoglycan in *L. lactis*. To test this hypothesis, we examined the cell morphologies of strains NZ9000 and NZ*rmaH* after 5 h of growth by SEM and TEM. As shown in Fig. 2A and B, no significant difference in cell surface morphologies of NZ9000 and NZ*rmaH* was observed by SEM, and both strains displayed regular coccoid shapes with smooth cell surfaces.

However, TEM observations showed distinct features in NZ*rmaH* cells (Fig. 2D). Some NZ*rmaH* cells displayed a white cell bulge on the inner side of the cell envelope (Fig. 2D, black open triangle), surrounded by a layer of undefined substances. In addition, obvious adhesion between adjacent NZ*rmaH* cells (Fig. 2D and F, white open triangle) is shown, potentially resulting from increased biosynthesis or secretion of cell surface viscous substances. The cell wall of both strains was clearly visible in TEM images (Fig. 2E and F, black arrow). ImageJ was used to measure the cell wall thickness (Fig. 2G). The average cell wall thickness of NZ*rmaH* was 24.17 ± 3.35 nm, which significantly increased by 52% (*P* < 0.001) than that of NZ9000 (15.90 ± 2.41 nm). Interestingly, the NZ9000 cell wall appeared more heavily stained with metal ions (lead citrate and uranyl acetate) than the NZ*rmaH* (Fig. 2E and F, black arrow). Consistent with a previous study where a similar staining pattern was observed between wild-type and *dltA* mutant strains of Group B *Streptococcus*, Saar-Dover et al. (27) believed that it was due to the decreased packing density of the *dltA* mutant cell wall. Therefore, we demonstrated that the overexpression of RmaH increased the cell wall thickness but might lead to a reduction in cell wall packing density.

### Transcriptional profiling of RmaH overexpression strain

To further study the regulatory function of RmaH in *L. lactis* and analyze its targets responsible for the acid tolerance, as well as the tolerance to nisin and vancomycin, we performed transcriptome analysis with strains NZ9000 and NZ*rmaH* harvested at the log phase (5 h, OD_600_ of approximately 0.7). A total of 755 genes, about 29.0% of the predicted genes in the *L. lactis* NZ9000 genome, were defined as differentially expressed genes (DEGs) (*P* < 0.05, |Log_2_foldchange [NZ*rmaH*/NZ9000]| ≥ 1.5) in the strain NZ*rmaH*. Among them, 390 genes were significantly upregulated (the number of gene transcripts was higher in NZ*rmaH*), and 365 genes were significantly downregulated, indicating that RmaH was a global transcription regulator. Then 23 genes (9 significantly upregulated genes, 4 genes without significant changes, and 8 significantly downregulated genes) were selected to verify their transcriptional levels by real-time fluorescence quantitative PCR (qRT-PCR) (Fig. S3). The qRT-PCR results showed a positive correlation with the RNA-seq data, suggesting the suitability of the transcriptome data for investigating the regulatory mechanisms involving the transcription factor RmaH. According to the Cluster of Orthologous Groups (COG) function classification, the significantly changed genes were broadly distributed in amino acid transport and metabolism, nucleotide transport and metabolism, carbohydrate transport and metabolism, cell wall/membrane/envelope biogenesis, and transcription (Fig. S4A).

Amino acid transport and metabolism were widely influenced by RmaH, including genes involved in arginine biosynthesis and metabolism, histidine biosynthesis, aromatic amino acid biosynthesis, branched-chain amino acid biosynthesis, and amino acid/oligopeptide transport (Fig. S5A, Table S3). Notably, the genes related to arginine biosynthesis (*argC*, *argE*, *argG*, and *argH*) were significantly activated by the overexpression of RmaH. Additionally, the transcriptional levels of arginine deamination pathway genes (*arcABC1C2D1T*) associated with the acid tolerance of bacteria were remarkably repressed in strain NZ*rmaH*, which was consistent with the negative role of RmaH in regulating acid tolerance of NZ9000.

Cell wall/membrane/envelope biogenesis was also found to be mediated by RmaH. The fatty acid biosynthesis and metabolism pathway was widely activated in NZ*rmaH* (Table S3). The transcript levels of genes involved in fatty acid and phospholipid biosynthesis (Fig. S5B), such as acetyl coenzyme A genes (*fabDFGHIZ1*), 1-acylglycerol-3-phosphate acyltransferase gene *plsC,* and phosphatidate cytidylyltransferase gene *cdsA*, were significantly increased; and the transcription of genes (*mvk* and *hmgA*) related to undecaprenyl phosphate (UP) embedded in cytoplasmic membrane was also activated. The alcohol dehydrogenase gene *adhA*, which participated in fatty acid degradation, was significantly downregulated. Moreover, the overexpression of RmaH promoted the transcription of the cell wall biosynthesis-associated genes (Table S3). The peptidoglycan biosynthesis genes (*murAA1B*, *murF*, *murI*, *ddl*, *dacA*, *murT*, *gatD,* and *uppS*), rhamnan polysaccharide biosynthesis (*rgpA*BCD), and D-alanylation of teichoic acid (TA) (*dltA*) were significantly activated in NZ*rmaH*. However, the transcription levels of genes *tagB*, *tagD,* and *tagF* involved in TA biosynthesis were decreased.

Carbohydrate transport and metabolism were also affected by RmaH (Fig. S5C, Table S3). In particular, the biosynthesis and metabolism of pyruvate were widely inhibited in NZ*rmaH*. The transcriptional levels of glycolysis pathway genes (*pgmB*, *pfkC*, *gapA,* and *pyk*) and pyruvate metabolism genes (*ldhB*, *ldh*, *als*, *butAB*, PDH complex, and *adhE*) were significantly downregulated. Furthermore, the nucleotide biosynthesis pathway was also widely influenced by RmaH (Fig. S5D, Table S3). The transcription of purine deoxynucleotide biosynthesis genes and pyrimidine deoxynucleotide biosynthesis genes was enhanced in NZ*rmaH*. Moreover, RmaH, as a global regulator, could regulate a series of transcription factors at the transcriptional level. The transcriptions of 27 putative regulators were affected by the overexpression of RmaH (Table S3). Overall, the transcriptome analysis revealed the potential function of RmaH, but the genes directly regulated by it need further identification.

### Identification of genes directly regulated by RmaH via ChIP-seq analysis

To identify the direct targets of the transcription factor RmaH, the ChIP-seq analysis was performed. The strain NZ*rmaH*-3Flag, which carried a plasmid expressing a 3Flag-tagged RmaH fusion protein, was cultured in GM17 medium until reaching the log phase and processed as described in Materials and Methods. A total of 632 peaks were screened (*q-*value < 0.05), of which 32.71% were located within the coding DNA sequence (CDS) region, and 66.96% were found in the promoter region (Fig. S4B). The genes associated with these binding regions were mainly enriched in carbohydrate metabolism, energy metabolism, amino acid metabolism, lipid metabolism, membrane transport, and nucleotide metabolism (Fig. S4D). This distribution aligned with the COG classification result from our transcriptome analysis. After combining the transcriptome and ChIP-seq analysis data, a total of 191 candidate targets directly regulated by RmaH (enrichment factor > 1.10 and |Log_2_fold change [NZ*rmaH*/NZ9000]| ≥1.5) were obtained (Fig. S4C, Table S4). Many of the genes that appeared in the transcriptome data, involved in amino acid transport and metabolism, fatty acid biosynthesis, nucleotide biosynthesis, and pyruvate metabolism, were found in ChIP-seq analysis as well, as shown in the boxes in Fig. S5.

**Fig 3:**
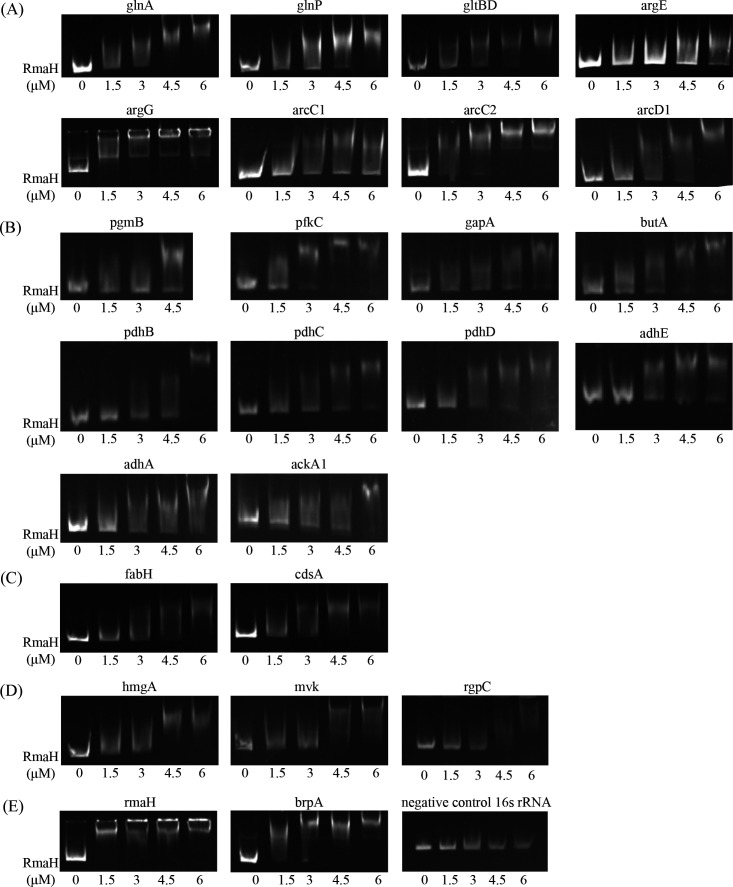
Detection of protein RmaH binding to target genes *in vitro*. (**A**) EMSA results of genes related to amino acid biosynthesis and transport; (**B**) EMSA results of genes related to pyruvate biosynthesis and metabolism; (**C and D**) EMSA results of genes related to cell wall and membrane biosynthesis; and (**E**) EMSA results of transcription factor genes and negative control.

The genes participating in arginine biosynthesis and metabolism (*argE*, *argG*, *arcC1*, *arcC2,* and *arcD1*), glutamate biosynthesis and transport (*glnA*, *gltBD,* and *glnP*), pyruvate biosynthesis and metabolism (*pgmB*, *pfkC*, *gapA*, *butA*, *pdhB*, *pdhC*, *pdhD*, *adhE*, *adhA,* and *ackA1*), fatty acid biosynthesis (*fabH* and *cdsA*), cell wall biosynthesis (*mvk*, *hmgA,* and *rgpC*), and transcription regulators (*brpA* and *rmaH*) were identified as direct targets of RmaH via the ChIP-seq. The direct binding of RmaH to the DNA regions of these 25 genes was verified by EMSA. The SDS-PAGE result of the purified His-tagged RmaH (18.6 kDa) is shown in Fig. S6. In contrast to the negative control, which used a random sequence from gene *16s rRNA*, clear band shifts were observed with all 25 candidate genes (Fig. 3). Additionally, RmaH exhibited a relatively higher affinity for the upstream region of its own gene (enrichment factor, 2.19-fold), indicating a self-regulation mechanism of RmaH. This observation was consistent with the known self-feedback regulation of the MarR family transcription factor (28).

### RmaH contributes to nisin and vancomycin tolerance by targeting cell wall biosynthesis genes

Modifications to the cell wall, such as thickening, increased density, and reduced electronegativity, are effective mechanisms to prevent nisin from reaching the cytoplasmic membrane and binding to the peptidoglycan precursor lipid II (29). Transcriptome analysis revealed that the transcriptional levels of cell wall peptidoglycan biosynthesis genes (*murA*, *murA1*, *murB*, *murF*, *ddl*, *ftsW1,* and *dacA*), D-alanylation of TA gene (*dltA*), and rhamnose biosynthesis genes (*rgpABCD)* were upregulated in strain *NZrmaH*. ChIP-seq analysis identified only *rgpC* as directly bound by RmaH, which was verified by EMSA. This suggested that the transcription factor RmaH might directly or indirectly activate the transcription of cell wall biosynthesis-associated genes, thus enhancing cell wall structure and improving the tolerance of strain NZ9000 to nisin or vancomycin.

**Fig 4:**
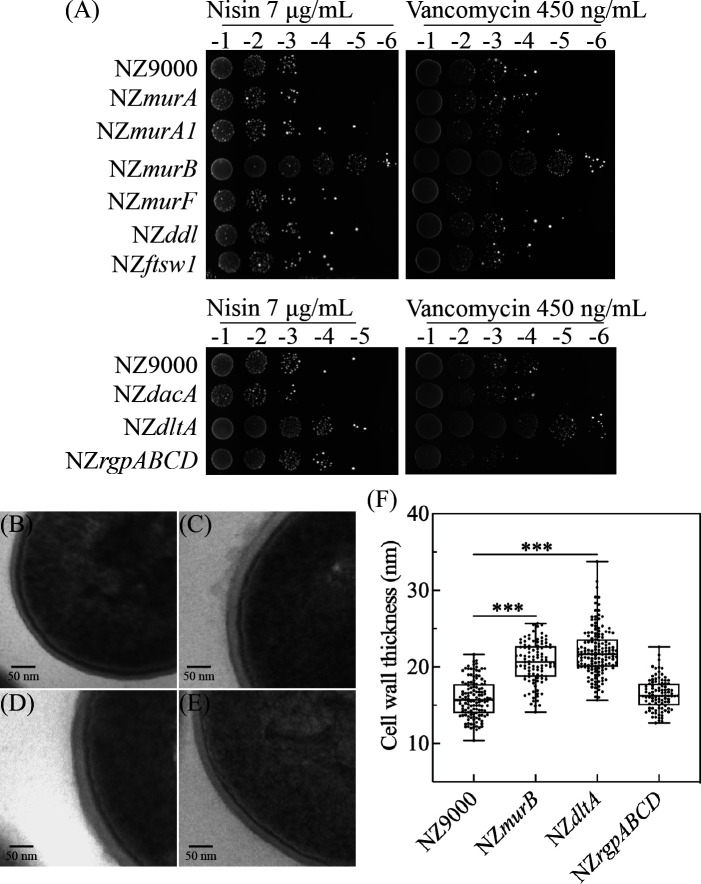
Effect of cell wall synthesis genes on nisin and vancomycin tolerance of *L. lactis*. (**A**) Nisin and vancomycin tolerance tests. TEM analysis of strains (**B**) NZ9000 and (**C**) NZ*murB*, (**D**) NZ*dltA* and (**E**) NZ*rgpABCD*. (**F**) Statistical analysis of cell wall thickness (***P < 0.001).

To validate the supposition, we constructed strains overexpressing the cell wall biosynthesis-associated genes and tested their antibiotic tolerance. As shown in Fig. 4, the strains overexpressing *murB* and *dltA* (NZ*murB* and NZ*dltA*) demonstrated significantly higher tolerance to both nisin and vancomycin, compared to NZ9000. The maximum valid dilutions for NZ*murB* and NZ*dltA* on the GM17 plate with 7 μg/mL nisin were 1:10^6^ and 1:10^5^, respectively, versus 1:10^3^ for NZ9000. For vancomycin, the maximum valid dilutions were 1:10^6^ for NZ*murB* and NZ*dltA* compared to 1:10^4^ for NZ9000. In addition, overexpression of *rgpABCD* also improved nisin tolerance, with NZ*rgpABCD* showing a maximum valid dilution of 1:10^4^. Combining the results of ChIP-seq analysis, these results indicated that RmaH likely activated the transcription of *rgpABCD* directly by binding to the *rgpC* CDS region and enhanced the transcription of *murB* and *dltA* indirectly, thereby improving the strain’s tolerance to nisin and vancomycin.

Then, TEM was used to assess the changes in cell wall morphology in the overexpressing strains NZ*murB*, NZ*dltA,* and NZ*rgpABCD*. All strains, including the wild-type NZ9000, exhibited normal cell envelope structures (Fig. 4B through E). Measurements of cell wall thickness using ImageJ (Fig. 4F) showed that NZ9000 cells had a mean cell wall thickness of 15.90 ± 2.41 nm. In contrast, the cell wall thickness of NZmurB and NZdltA cells increased significantly (*P* < 0.001) to 20.54 ± 2.70 and 21.95 ± 3.40 nm, respectively, reflecting increases of 29.2% and 38.1%. The thickness of strain NZ*rgpABCD* was 16.54 ± 2.40 nm, comparable to the wild type. Combined with the result that RmaH positively regulated the cell wall thickness (Fig. 2G), it suggests that RmaH could activate the transcription of *murB* and *dltA* to increase the cell wall thickness. In summary, this indicates that RmaH increased the cell wall thickness of NZ9000 by indirectly activating the transcription of *murB* and *dltA*, which contributed to enhanced tolerance of the strain to nisin and vancomycin.

### RmaH contributes to nisin and vancomycin tolerance by targeting membrane lipid biosynthesis genes

The composition, integrity, and fluidity of the cell membrane are crucial for bacteria’s adaptation to diverse environmental stresses, including exposure to low pH, suboptimal temperature, and antibiotics (29–31). Transcriptome data showed that the transcription levels of fatty acid biosynthesis genes (*accB*, *accC1,* and *fabDFGHIZ1*), membrane lipid synthesis genes (*fatA*, *plsC*, *clsA*, *cdsA,* and *dgk*), and UPP synthesis genes (*hmgA* and *mvk*) were significantly upregulated in strain NZ*rmaH*. These genes were assumed to be potential contributors to the enhanced tolerance of NZ9000 to nisin and vancomycin. Consequently, we engineered overexpression strains of the 15 genes related to membrane lipid biosynthesis and tested their impact on the strain’s nisin and vancomycin tolerance.

**Fig 5:**
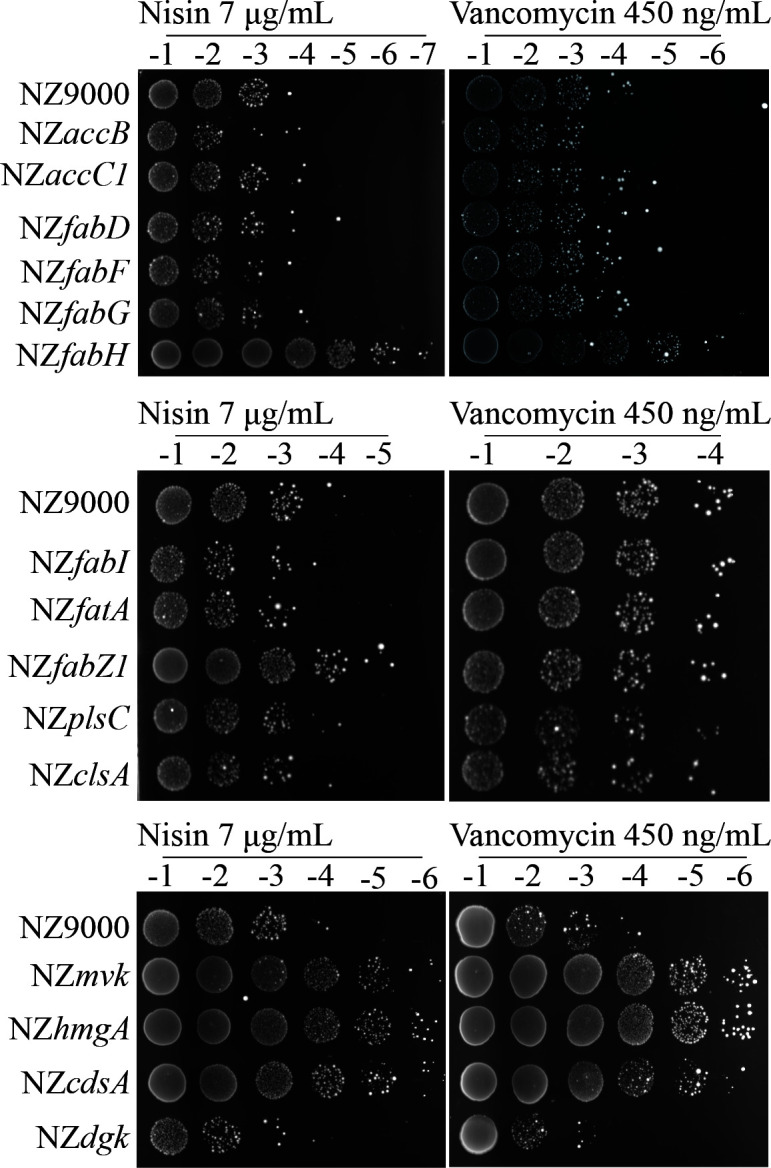
Nisin and vancomycin tolerance of *L. lactis*. The experimental conditions are shown in “Lactic acid tolerance, nisin tolerance, and vancomycin tolerance assays”.

As shown in Fig. 5, the tolerances of the strains overexpressing *fabH*, *cdsA*, *hmgA,* and *mvk* (named NZ*fabH*, NZ*cdsA*, NZ*hmgA,* and NZ*mvk*) exhibited significantly improved tolerance to nisin and vancomycin compared to NZ9000 (the maximum valid dilutions: 1:10^3^ for nisin and 1:10^4^ for vancomycin). Specifically, on GM17 plates containing 7 μg/mL nisin, strains NZ*fabH*, NZ*cdsA*, NZ*hmgA,* and NZ*mvk* achieved maximum valid dilutions of 1:10^7^, 1:10^6^, 1:10^6^, and 1:10^6^, respectively. Similarly, on the GM17 plate with 450 ng/mL vancomycin, all engineered strains maintained maximum valid dilution of 1:10^6^. Additionally, the nisin tolerance of strain NZ*fabZ1* was also increased more than 10-fold. Furthermore, the ChIP-seq and EMSA results confirmed that RmaH directly bound to the CDS region of *fabH*, *cdsA*, *mvk,* and the promoter region of *hmgA*. In general, these findings underscore the role of RmaH in augmenting the tolerance of NZ9000 to nisin and vancomycin through direct activation of critical genes involved in membrane lipid biosynthesis, namely *fabH*, *cdsA*, *mvk,* and *hmgA*.

### RmaH negatively controls the pHi

Although RmaH positively regulates bacterial tolerance to nisin and vancomycin, its overexpression was detrimental to bacterial acid tolerance. Combining the transcriptome analysis, ChIP-seq analysis, and the EMSA results, the arginine biosynthesis genes (*argE* and *argG*) and ADI pathway-related genes (*arcC1*, *arcC2,* and *arcD1*) were upregulated and downregulated in NZ*rmaH*, respectively, and the five genes could be directly bound to RmaH. The ADI pathway plays a key role in the acid tolerance of LAB via mediating the concentration of intracellular H^+^. Consequently, the intracellular pH (pHi) value was detected. As shown in Fig. 1G, the pHi value of NZ*rmaH* (6.18 ± 0.03) was significantly lower than that of the wild-type strain NZ9000 (6.45 ± 0.05, *P* < 0.001), suggesting a negative role of RmaH in regulating pHi. These results indicated that RmaH could decrease the pHi by directly inhibiting the transcriptions of ADI pathway-related genes *arcC1*, *arcC2,* and *arcD1*, thereby weakening the adaptability of *L. lactis* NZ9000 to acid stress.

### Characterization of the DNA motif bound by RmaH

**Fig 6:**
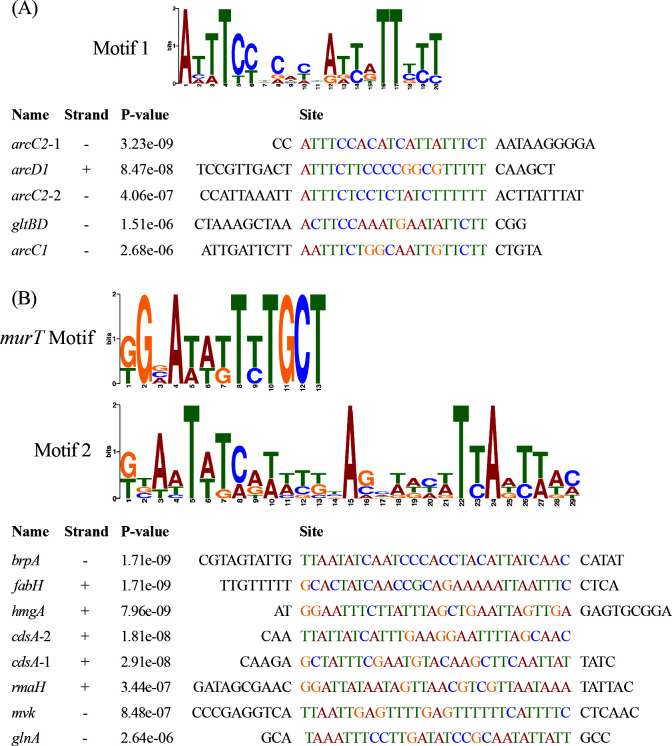
Prediction of the binding motif of RmaH to DNA. (**A**) DNA motif prediction of inhibited targets and (**B**) DNA motif prediction of activated targets.

The above investigation indicated direct binding of RmaH to genes involved in amino acid metabolism (*arcC1*, *arcC2*, *arcD1*, *glnA,* and *gltBD*), membrane lipid biosynthesis (*fabH*, *cdsA*, *mvk,* and *hmgA*), and transcriptional regulation (*rmaH* and *brpA*). Due to the extensive sequence range from the ChIP-seq data, EMSA with truncated sequences was performed to limit the sequence length to 30–50 bp. The detailed processes for the 11 genes are illustrated in Fig. S7 to S9, and the specific binding sequences of RmaH to the 11 genes are outlined in Table 1. A MEME analysis on the specific binding sequences of the 11 genes revealed two conserved AT-rich motifs (Fig. 6). Motif 1, “ATTTCYWCAYHATTRTTYTT,” was conserved in the suppressed targets *arcC1*, *arcC2*, *arcD1,* and *gltBD*, exhibiting symmetrically distributed T bases at both ends. The presence of this motif within the CDS of the downregulated genes suggested that RmaH likely acted as a negative regulator via a transcriptional roadblock mechanism, thereby inducing premature termination (32).

**TABLE 1:** DNA binding sequences of RmaH and the target genes

Gene	DNA sequences (5′−3′)	Position relative to the starting codon of the gene (bp)	CDS/intergenic
Inhibition			
*arcC1*	TACAGAAGAACAATTGCCAGAAATTAAGAATCAATTTCC	+443	ORF
*arcC2*-1	CAATTAATTCCCCTTATTAGAAATAATGATGTGGAAATGG	−9	Intergenic
*arcC2*-2	CCATAAATAAGTAAAAAAGATAGAGGAGAAATAATTTAATGG	+135	ORF
*arcD1*	GACTTCCGTTGACTATTTCTTCCCCGGCGTTTTTCAAGCT	+333	ORF
*gltBD*	CCGAAGAATATTCATTTGGAAGTTTAGCTTTAGTTG	+3278	ORF
Activation			
*glnA*	GGCAATAATATTGCGGATATCAAGGAAATTTATGC	−207	Intergenic
*fabH*	TTGTTTTTGCACTATCAACCGCAGAAAAATTAATTTCCTCA	+365	ORF
*cdsA*-1	CAAGAGCTATTTCGAATGTACAAGCTTCAATTATTATC	+158	ORF
*cdsA*-2	CAATTATTATCATTTGAAGGAATTTTAGCAAC	+133	ORF
*mvk*	GTTGAGGAAAATGAAAAAACTCAAAACTCAATTAATGACCTCGGGCAATTAGCC	+620	ORF
*hmgA*	ATGGAATTTCTTATTTAGCTGAATTAGTTGAGAGTGCGGA	−416	Intergenic
*rmaH*	CTTGACAAAAGATAGCGAACGGATTATAATAGTTAACGTCGTTAATAAATATTAC	−38	Intergenic
*brpA*	ATATGGTTGATAATGTAGGTGGGATTGATATTAACAATACTACGG	+536	ORF

Motif 2 (“KBAWTWTCAWWTKYA-SMWDHWTTADTWWM”), identified in the activated targets *glnA*, *fabH*, *cdsA*, *hmgA*, *mvk*, *rmaH,* and *brpA*, shared similarity with the reported DNA motif of *murT* (26). A sequence “GTAATATCATTTTYAGMTACTTTAATTAC” could be obtained by selecting the base with the maximum probability of occurrence. It contained a central, fully conserved base A and exhibited an imperfect palindromic symmetry, consistent with the recognition of AT-rich palindromes by MarR-family proteins. These findings aligned with previous observations that MarR family proteins recognized AT-rich palindrome sequences; for example, *R. jostii* RHA1 CouR identified “cATTGAnnnnnTCAATg” (8), while *R. palustris* CouR acknowledged “GTTATAnnnTATAAC” (9). RmaH could bind to the promoter regions of genes *murT* (26), *glnA*, *hmgA,* and *rmaH* to stimulate transcription through DNA conformational changes or direct interaction with RNA polymerase subunits α and σ (33). Intriguingly, activation of *fabH*, *cdsA*, *mvk,* and *brpA* occurred through direct RmaH binding to their CDS, extending the paradigm established for *E. coli* global regulators, such as Cra (34), RutR (35), CRP (36), LeuO (37), and NtrC (38). This CDS-mediated activation might function as an anti-silencing mechanism (39, 40), wherein RmaH competitively displaced the H-NS protein, which repressed transcription by binding open reading frames (ORFs) and promoting termination (41). However, limited in-depth studies have focused on the ORF-bound transcription factors activating transcription, underscoring the need for mechanistic studies on ORF-bound transcription factors.

### Characterization of key amino acids of RmaH involved in binding to the DNA sequence

The amino acid sequences of RmaH and 16 homologous proteins were aligned for comparison and analysis of conserved amino acids. Pfam and Phyre2 were used to predict the conserved domain and secondary structure of RmaH (Fig. S10). RmaH possessed a typical HTH domain structured as α2-α3-α4-β1-β2, highly conserved in the genus *Lactococcus*. In addition, the amino acid residues I53, A54, L71, R79, D85, R87, T94, and G97, located in the HTH domain of RmaH, were completely conserved in the 17 proteins, suggesting that the 7 amino acid residues (I53, L71, R79, D85, R87, T94, and G97) might play important roles in the binding of RmaH to target DNA. To verify this speculation, the seven amino acid residues were replaced with alanine (A) using site-directed mutagenesis. The resulting mutant proteins were denoted as I53A, L71A, R79A, D85A, R87A, T94A, and G97A, respectively, and the SDS-PAGE result of the purified His-tagged mutant proteins (molecular weight, 18.6 kDa) is shown in Fig. S6.

**Fig 7:**
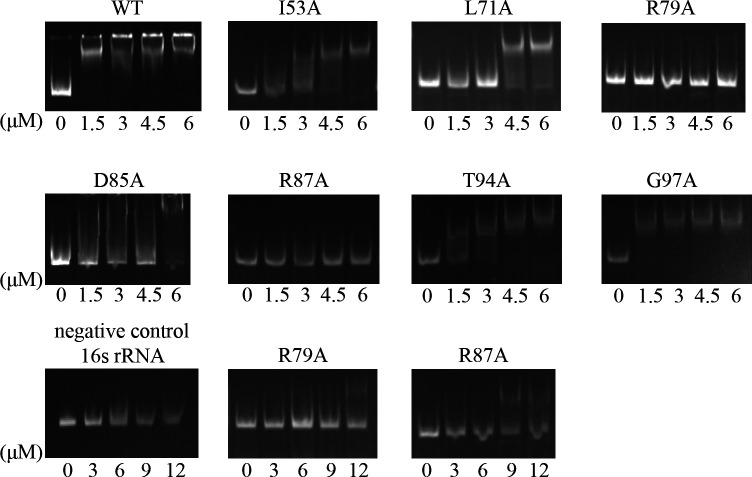
EMSA results of RmaH and its mutants binding to the DNA-binding site of the gene *rmaH*. The gel blots of “WT” and “16s rRNA” are identical to those of “rmaH” and “negative control 16s rRNA” in Fig. 3E, respectively, serving as controls for comparative purposes.

EMSA was used to confirm the binding of the identified promoter region of *rmaH* (P*rmaH*) by the seven mutant proteins (Fig. 7). Proteins T94A and G97A exhibited nearly identical binding capabilities to P*rmaH* compared to RmaH, while the binding abilities of the mutant proteins I53A, L71A, D85A, R79A, and R87A to P*rmaH* were weakened, particularly R79A and R87A. Subsequently, a microscale thermophoresis (MST) assay was performed to test the binding kinetic constants of protein RmaH and its mutants with P*rmaH*. In keeping with the EMSA results, the binding affinities of I53A, L71A, D85A, R79A, and R87A to P*rmaH* decreased, as evidenced by the increased equilibrium dissociation constant (KD) (Table 2 ; Fig. S11). In particular, the binding affinity of the wild type was approximately 13.6- and 9.4-fold higher than that of R79A and R87A, respectively. These results were consistent with the findings on MarR family proteins OhrR (42), MarR (43), MexR (44), and SarR (45), which demonstrated that arginine residues in the β1 and β2 folds were crucial for binding to target DNA *in vitro*. Additionally, the Asp residue also played an important role in binding. In *S. aureus* MepR, Asp85 and Arg87 formed hydrogen bonds with the adenine and thymine bases in the minor groove of DNA (10). Similarly, in *P. aeruginosa* MexR, hydrophobic α3/α4-helix interactions stabilized dimerization, while surface Arg residues in the β-turn enhanced DNA affinity via electrostatic forces (46). Thus, we proposed that RmaH dimer stability involved I53 (α3-helix) and L71 (α4-helix), whereas D85, R79, and R87 in β-turns—particularly R79 and R87—were critical for DNA binding.

**TABLE 2:** The KD values for the binding of RmaH and its mutants to the DNA sequence

Protein	KD (μM)	KD confidence
WT	1.09	2.76E-07
I53A	9.20	4.96E-06
L71A	7.67	9.221E-07
R79A	14.81	8.65E-06
D85A	6.10	2.79E-06
R87A	10.26	5.53E-05
T94A	2.02	5.69E-07
G97A	0.91	1.49E-07

## DISCUSSION

**Fig 8:**
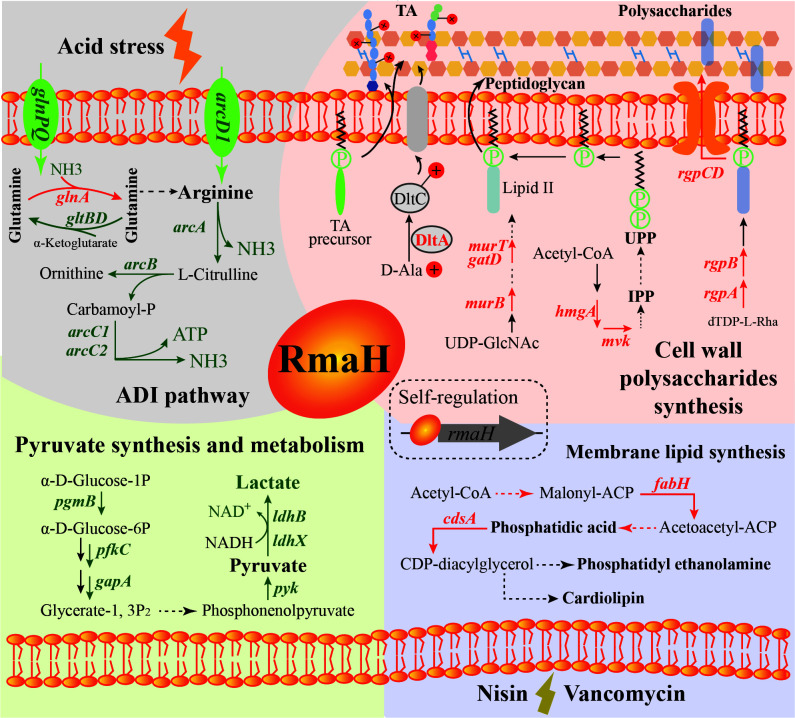
The proposed mechanism of RmaH regulating the acid stress and the tolerance to nisin and vancomycin of *L. lactis*. Genes in red indicate that the transcriptional level is activated by RmaH, whereas green indicates inhibition.

In this study, we identified the MarR family regulator RmaH as a key modulator of *L. lactis* tolerance to the antimicrobial peptide nisin, the antibiotic vancomycin, and acidic environments. Transcriptome and ChIP-seq analysis revealed RmaH to be a global transcription factor affecting various pathways, such as amino acid transport and metabolism, cell wall/membrane/envelope biogenesis, carbohydrate transport and metabolism, and nucleotide transport and metabolism. Through investigation of RmaH targets, we elucidated the mechanisms by which RmaH regulated the tolerance to acid, nisin, and vancomycin of *L. lactis*. The regulation mechanism mediated by RmaH is summarized in Fig. 8. Furthermore, at the molecular level, the DNA-binding motifs and key amino acids involved in RmaH binding to its target genes were identified.

Increasing cell wall thickness and D-alanylation of teichoic acid play important roles in bacterial tolerance to nisin and vancomycin (47–49). The lipid carrier UP, embedded in the membrane, is essential for transporting precursors of major cell wall components, including peptidoglycan and teichoic acids (50). Its biosynthesis depends on the mevalonate pathway (51), where HmgA and Mvk serve as critical enzymes, with HmgA being rate-limiting (52). We found that overexpression of *hmgA* and *mvk* significantly improved the tolerance to nisin and vancomycin. Furthermore, upregulating *murB* and *dltA* also enhanced antibiotic tolerance by promoting cell wall thickening. Although overexpression of other individual cell wall biosynthesis genes did not significantly affect tolerance, combinatorial effects might exist. Molecular evidence from ChIP-seq and EMSA indicated that RmaH directly activated the transcription of *hmgA* and *mvk*, while it indirectly regulated *murB* and *dltA*, potentially through an unidentified secondary transcriptional regulator (Fig. S12). In conclusion, we proposed that RmaH fortified the cell wall by directly stimulating precursor supply via the mevalonate pathway and indirectly modulating specific biosynthetic genes, thereby increasing nisin and vancomycin tolerance in *L. lactis* NZ9000. In our recent work, we have identified RmaH as an activator of the peptidoglycan biosynthesis operon *murT-gatD* (26); however, this regulation was closely related to the acid tolerance of *L. lactis*, which paradoxically reduces acid tolerance by altering peptidoglycan charge. These two related works suggested that RmaH coordinated cell wall remodeling in response to different environmental stresses, though with potentially contrasting physiological outcomes. It also highlighted a possible trade-off between antibiotic tolerance and acid tolerance mediated by RmaH based on cell wall remodeling.

Bacterial membrane phospholipid biosynthesis relies on the conserved type II fatty acid synthesis (FAS II) pathway (53). β-ketoacyl-(acyl carrier protein) synthase III (FabH) initiates FAS II by catalyzing the first elongation cycle (54). FabH is crucial for membrane integrity. The *E. coli* Δ*fabH* strain showed drastically increased sensitivity to vancomycin and other antibiotics (55), accompanied by reduced saturated fatty acid content and slower envelope growth (56). Conversely, increasing FabH expression in *Rhodobacter sphaeroides* Rs-A2 boosted long-chain fatty acid production (57). In *L. lactis* IL1403, FabH was also essential for growth, as mutants failed to grow without exogenous long-chain fatty acids (58). Based on these findings, we assumed that RmaH-mediated activation of *fabH* transcription altered the cytoplasmic membrane composition, enhancing *L. lactis* NZ9000 tolerance to nisin and vancomycin.

The core metabolism of *L. lactis* is characterized by its reliance on glycolysis for ATP generation and the dominant conversion of pyruvate to lactate. This homolactic fermentation is crucial for redox balance, as lactate dehydrogenase regenerates NAD+ by consuming the NADH produced during glycolysis (59). In this study, the coordinated downregulation of both the EMP pathway genes (*pgmB*, *pfkC*, and *gapA*) and *ldhB* in the NZ*rmaH* strain might constitute a specific physiological response to reallocate cellular resources. By attenuating both the production and consumption of glycolytic NADH in tandem, the cell established a new redox balance at a lower flux level. The conserved energy and metabolic precursors might thereby be redirected toward RmaH-activated biosynthesis of bacterial defense structures, such as the thickened cell wall, which prioritize stress resistance over rapid growth.

An intriguing finding of our study was the trade-off mediated by RmaH, whereby enhanced tolerance to nisin and vancomycin was achieved by diminished acid resistance. We proposed that this seemingly counterproductive regulation might reflect a sophisticated ecological strategy to optimize fitness within a competitive and dynamic niche. From the perspective of microbial competition and resource allocation, this regulation provided a decisive advantage. During initial colonization, rapid environmental acidification, supported by robust acid production and tolerance, is vital. Repression of RmaH enabled cells to prioritize resource investment toward acidification, thereby inhibiting competitors and securing a dominant position in the ecological niche. This “acidic blitzkrieg” facilitated rapid nutrient consumption and population expansion. However, the resulting high-density, nutrient-depleted, and acidic environment led to subsequent challenges, including accumulation of self-generated lactic acid and exposure to bacteriocins or antibiotics released from neighboring cells. Under these conditions, constitutive investment in extreme acid resistance became less advantageous. Instead, RmaH activation triggered a strategic reallocation of finite cellular resources, reinforcing the cell wall and membrane. This shift represented a transition from an offensive growth strategy to a defensive survival posture. The fortified cell envelope served as a broad-spectrum barrier against rising external threats, such as nisin and vancomycin. Thus, RmaH enabled cells to dynamically manage their resource, ensuring not only transient growth superiority but also long-term persistence and survival under evolving environmental pressures.

In addition, RmaH belongs to the MarR family of transcriptional regulators, many members of which are known to bind specific cofactors (such as metabolites or small molecules) that alleviate regulator-DNA binding (8, 60, 61). In this study, we successfully mapped the regulatory network of RmaH by identifying its targets based on an overexpression strategy. However, whether physiological cofactors that affect the binding of RmaH to its targets exist, and how these cofactors participate in its functional regulation, remain unanswered questions of great exploratory value. Future investigations aimed at identifying these potential cofactors will be essential to fully elucidate the mechanistic role of RmaH in *L. lactis*.

In conclusion, our study elucidated the multifaceted role of the MarR-family regulator RmaH in *L. lactis*. We have mechanistically demonstrated that RmaH, through direct transcriptional control, enhanced antibiotic tolerance by activating cell wall and membrane biosynthesis while simultaneously repressing acid resistance pathways. This regulatory trade-off might reflect an optimal resource allocation strategy for sequential environmental challenges. The molecular basis of this regulation was defined by specific DNA motifs and key arginine residues (R79 and R87). Collectively, these findings provided a comprehensive understanding of how a single transcriptional factor integrated multiple environmental signals to coordinate an adaptive response that optimized fitness in a complex and competitive ecosystem.

## MATERIALS AND METHODS

### Strains and growth conditions

The strains used in this study are shown in Table S1. *L. lactis* NZ9000 was cultured in GM17 broth (M17 broth supplemented with 0.5% (wt/vol) glucose at 30°C under static conditions. *E. coli* TG1 was used as the host for cloning, and *E. coli* BL21 was used for protein expression. For the selection of *E. coli* transformants, LB medium was supplemented with either 200 μg/mL erythromycin (Em) or 100 μg/mL kanamycin (Kana). Recombinant *L. lactis* was selected using growth medium containing 10 μg/mL Em.

### Plasmids and recombinant strain construction

Primers are shown in Table S2. The pLEB124- and pET28a-derived plasmids shown in Table S1 were constructed by the seamless cloning technology. The plasmid pLEB124 with a constitutive promoter P_45_, used for overexpressing gene *rmaH*, was amplified by PCR to obtain a linearized plasmid and purified with a DNA gel extraction kit (Tiangen Biotech, Beijing, China). The target gene *rmaH* fragments carrying ~20 bp arm sequences identical to the linearized plasmid pLEB124 on both sides were amplified, purified, and cloned into the linearized pLEB124 using the Minerva Super Fusion Cloning Kit (US Everbright Inc.), resulting in the recombinant plasmid pLEB124-*rmaH*. The plasmid pLEB124-*rmaH* was then transformed into *L. lactis* NZ9000 through electroporation to gain the *rmaH-*overexpressing *L. lactis* strain NZ*rmaH* (26). With the same method, the gene overexpression strains related to cell wall peptidoglycan biosynthesis and fatty acid biosynthesis were constructed. The pET28a-derived vectors were also constructed in the same way and then transformed into *E. coli* BL21 for protein expression and purification.

### Determination of CFU

For growth analysis, the activated *L. lactis* strains were inoculated at 1% (vol/vol) into 50 mL of GM17 medium. Samples were collected at 2 h intervals to determine the viable count. Aliquots of each sample were serially diluted in sterile saline, and 0.1 mL of the appropriate dilution was spread onto GM17 agar plates. The plates with 30–300 discrete colonies were selected for enumeration. The CFU in 1 mL of bacterial solution was calculated using the formula: CFU/mL = (number of colonies × dilution factor)/volume plated (in milliliters).

### Lactic acid tolerance, nisin tolerance, and vancomycin tolerance assays

Strains of *L. lactis* grown for 5 h were used for the stress tolerance tests. The lactic acid tolerance and nisin tolerance assays were carried out according to previous studies (22, 26). For the lactic acid tolerance assay, the 5-h-grown cells of different strains were harvested, resuspended in GM17 medium adjusted to pH 4.0 with lactic acid at an identical OD_600_, and incubated statically for 3 h at 30°C. After the 3-h lactic acid stress, the suspension was serially diluted, and 8 µL of each dilution was spotted onto the GM17 plates. For the nisin tolerance and vancomycin tolerance assays, the cells of different strains grown for 5 h were resuspended in 0.9% NaCl at an identical OD_600_. Then, the suspension was serially diluted, and 8 µL of each dilution was spotted onto the GM17 plates with 8 μg/mL nisin or 450 ng/mL vancomycin. The number of viable cells was analyzed after 36 h of incubation. The bacterial suspension samples used for antibiotic/acid stress experiments, whose OD_600_ was adjusted to the same value, were diluted in a gradient and plated onto GM17 plates without antibiotics to test whether the biomass was consistent (results shown in Fig. S2).

### Real-time fluorescence quantitative PCR

To test the accuracy of the transcriptome data, the strains grown in the same conditions and the same RNA extraction method were applied to ensure the consistency of the experiment. The strains *L. lactis* NZ9000 and NZΔ*lssR* grown for 5 h were used for RNA extraction with RNAprep pure Cell/Bacteria Kit (Tiangen Biotech Co., Ltd.) following the instructions. The cDNA used for qRT-PCR was obtained by reverse transcription using SPARKscript II RT Master Mix (SparkJade). qRT-PCR was performed with 2× SYBR Green Fast qPCR Mix (BioMarker) on a QuantStudio 3 Real-Time PCR System (Applied Biosystems, USA). The 16S rRNA was used as a reference gene, and the relative transcriptional levels of targets were calculated with -∆∆CT (62). The concentration of cDNA used as the template for 16S rRNA amplification was diluted 1,000-fold compared to that used for the target gene. Three independent experiments were performed. The value of |∆∆CT| ≥ 1.5 is considered a significant difference.

To further verify whether the expression of the genes involved in the ADI pathway and cell surface polysaccharide biosynthesis in the strain NZΔ*lssR* also responded to the acid and nisin stresses, *L. lactis* NZ9000 and NZΔ*lssR* grown for 5 h were harvested and exposed to GM17 medium acidified with HCl (pH 3.0), lactic acid (pH 4.0), and containing 7 μg/mL nisin, respectively, for 3 h at 30°C. The total RNAs were extracted from the above strains for qRT-PCR with the same approach as described above.

### Cell morphology analysis

*L. lactis*, grown to the mid-exponential phase (~5 h), was collected for the cell morphology analysis by SEM and TEM. The samples for SEM observation were treated as described in the literature (63). The collected cells were washed three times with PBS buffer and fixed in 2.5% glutaraldehyde overnight at 4°C. After three washes with PBS buffer, the pellets were dehydrated with ethanol of gradient concentrations (50%, 70%, 80%, 90%, and 100%), lyophilized, and prepared for SEM (Hitachi High-Technologies, Tokyo, Japan) observation.

The preparation for the TEM analysis was performed as described previously (64). *L. lactis* strains were harvested, washed with PBS buffer, and then fixed in 2.5% glutaraldehyde overnight at 4°C. Cells were washed with PBS buffer and fixed in 1% osmium tetroxide for 1–2 h. Samples were then washed three times to remove the osmium tetroxide, dehydrated with ethanol at different concentrations (50%, 70%, 80%, 90%, and 100%), and embedded in Eponate 12 resin overnight at 70°C. Sections of 70–90 nm were prepared and stained with uranyl acetate and lead citrate for 5–10 min, respectively. TEM (HITACHI H-7650, Japan) was used to observe morphology.

### RNA extraction, sequencing, and transcriptome analysis

The strains NZ9000 and NZ*rmaH,* grown for 5 h (OD_600_ of approximately 0.7), were collected for the total RNA extraction using RNAprep pure Cell/Bacteria Kit (Tiangen Biotech Co., Ltd.) in accordance with the manufacturer’s instructions. Library construction, RNA sequencing (RNA-seq), and transcriptome analysis were conducted by Personal Biotechnology Co., Ltd. (Shenzhen, China). Libraries with insert sizes of 300–400 bp were prepared, and the quality of the sample was examined using an Agilent 2100 Bioanalyzer (Agilent Technologies, Santa Clara, CA, USA) and sequenced on the HiSeq 4000 System (TruSeq SBS KIT-HS V3, Illumina). Raw data primarily produced by Illumina HiSeq TM 4000 were quality controlled by analyzing the base composition and quality distribution of the bases along the reads (Q20 > 95% and Q30 > 90%) to ensure accuracy. DEGs were identified with a *P* value cutoff of 0.05 and |log2-fold change(NZ*rmaH*/NZ9000)| ≥ 1.5 in DESeq and classified based on the Cluster of Orthologous Groups classification (https://www.ncbi.nlm.nih.gov/COG/). The RNA-seq data of *L. lactis* NZ9000 and NZ*rmaH* obtained in this study were deposited in the NCBI Sequence Read Archive under BioProject number PRJNA770859.

### Chromatin immunoprecipitation sequencing

ChIP-seq was performed and sequenced following the previous protocol (45) by Wuhan IGENEBOOK Biotechnology Co., Ltd. The process is briefly described as follows. *L. lactis* cells were crosslinked with 1% formaldehyde in GM17 medium for 20 min, and 125 mM glycine was added to stop the crosslinking. The pellets were collected, washed with 1× PBS containing 1 mM proteinase inhibitor three times, and then resuspended in nuclei lysis buffer for 30 min. The chromatin was sonicated to sizes of 200–500 bp and incubated with 10 μg anti-Flag antibody (MAB 3118) for 16 h at 4°C, then 30 μL of protein G magnetic beads (Life Technologies) was added and incubated at 4°C for 2 h with rotation. After being washed with low salt wash buffer, high salt wash buffer, LiCl wash buffer, and TE buffer, the immunoprecipitate was eluted with elution buffer at 65°C for 20 min. The DNA was released by the addition of Proteinase K to digest the proteins and purified by phenol/chloroform/isoamyl extraction.

The DNA fragments were amplified by PCR for 15 cycles following repair and adaptor ligation steps. Libraries were validated on the Bioanalyzer 2100 (Agilent) and Qubit fluorometer (Invitrogen, Carlsbad, CA, USA). The ChIP-seq libraries were sequenced using the HiSeq 2000 system (Illumina) for 150 nt double-end sequencing. The wild-type NZ9000 and the strain overexpressing RmaH-3Flag were set as Input and IP, respectively. The significant peak was selected with a *q-*value < 0.05. The ChIP-seq data of *L. lactis* NZ9000 and NZ*rmaH*-3Flag obtained in this study were deposited in NCBI Sequence Read Archive under BioProject number PRJNA770371.

### Protein expression and purification

The protein-expressing strain was grown in LB medium with Kana to an OD_600_ of 0.6, and 0.1 mM isopropyl-β-D-thiogalactoside was then added to induce protein expression at 16°C for 16 h. Cells were suspended in 30 mL of lysis buffer (50 mM Tris-HCl [pH 8.0] and 0.3 M NaCl) and lysed by sonication for 30 min. The supernatant was collected after centrifugation (12,000 rpm) for 30 min and used for protein purification by Ni^2+^ affinity column. The protein was eluted by a linear gradient using elution buffer (50 mM Tris-HCl [pH 8.0], 0.3 M NaCl, and 300 mM imidazole) and further purified by desalination column PD10 (GE Healthcare, USA). Purified protein was stored in 10% glycerol at −80°C.

### Electrophoretic mobility shift assay

EMSA was conducted as described in a previous study (26). Briefly, DNA fragments labeled with fluorescence Cy5 (50 ng) were incubated with the purified protein in different concentrations at 25°C for 15 min in a 20 μL reaction system containing 4 μL of 5× EMSA buffer (100 mM Tris, 0.2 mg mL^−1^ BSA, 25% glycerol, 50 mM MgCl_2_, and 1 mM DTT) and 1 μg ssDNA. After pre-run for 30 min in 0.5× Tris-Borate-EDTA buffer, samples were loaded onto an 8% native polyacrylamide gel for another 15 min on ice water at 100 V. Bands were visualized with a fluorescence detecting system (Tanon-5200Multi, China).

### Determination of intracellular pH

The pHi was measured with the BBcellProbe BCE Kit in a fluorescence method (22). Cells were collected, washed twice, and resuspended in PBS buffer containing fluorescent probes for 30–60 min. The calibration curve was determined using a series of pH standard solutions at 5.5, 6.0, 6.5, 7.0, and 7.5. The harvested cells were resuspended in different pH standard solutions with fluorescent probes, respectively, and then 1 μM valinomycin and nigericin were added at 30°C for 10 min. The fluorescence microplate reader (Thermo Scientific Varioskan LUX, USA) was used to measure the fluorescence signal with its excitation and emission wavelengths at 488 and 528 nm, respectively.

### MEME motif prediction

The motif-based sequence analysis tool MEME (https://meme-suite.org/meme/) (1) was utilized to predict the DNA motif sequences of target genes *arcC1*, *arcC2*, *arcD1*, *gltBD*, *glnA*, *fabH*, *cdsA*, *hmgA*, *mvk*, *rmaH,* and *brpA*. The binding site search was conducted using the DNA sequences presented in Table 1, which were obtained through EMSA.

### Multiple sequence alignment

A Basic Local Alignment Search Tool (BLAST, https://blast.ncbi.nlm.nih.gov/Blast.cgi) search using the RmaH protein sequence was done. The homologous proteins in the other species of *Lactococcus* (excluding *L. lactis*), which showed the best match in BLAST, and three homologous proteins from other bacterial genera identified in the PDB database, were selected for multiple sequence alignment. Multiple sequence alignment of RmaH and its homologous proteins was generated by CLUSTALW (https://www.genome.jp/tools-bin/clustalw) and visualized in ESPript 3.0 (https://espript.ibcp.fr/ESPript/cgi-bin/ESPript.cgi). RmaH, WP_153496607.1 (*Lactococcus hircilactis*), WP_096817042.1 (*Lactococcus fujiensis*), WP_120771914.1 (*Lactococcus allomyrinae*), WP_142767004.1 (*Lactococcus* sp. KACC 19320), WP_109245609.1 (*Lactococcus termiticola*), PCS19903.1 (*Lactococcus tructae*), WP_200406721.1 (*Lactococcus taiwanensis*), WP_130123197.1 (*Lactococcus* sp. S-13), WP_068162537.1 (*Lactobacillus plantarum*), WP_198493992.1 (*Lactococcus garvieae*), KXT62389.1 (*Lactococcus* sp. DD01), WP_213534944.1 (*Lactococcus nasutitermitis*), MQW23638.1 (*Lactococcus* sp*. dk101*), 3OOP (*Listeria innocua Clip*11262), 2ETH (*Thermotoga maritima*), and 5FFX (*Staphylococcus aureus*) were applied. The conserved domain of protein RmaH can be predicted by the Pfam Database (http://pfam.xfam.org/). Secondary structure prediction of RmaH was carried out by Phyre version 2.0 (http://www.sbg.bio.ic.ac.uk/phyre2/). The RmaH protein sequence was entered on the website to conduct the Phyre search. The spatial structure of the target sequence can be predicted by identifying the folding types similar to the target sequence from the protein structure database.

### Microscale thermophoresis

MST was used to determine the apparent KD values of LssR and *hddA*, *hddA*3, and *hddA*6, respectively. Three hundred nanomolar DNA labeled with fluorescence Cy5 was mixed with increasing LssR concentrations using a twofold dilution method. The mixture was filled into the capillary pipette, and then the distribution of molecules in the temperature gradient field was measured by monitoring the distribution of fluorescence signals in MST (Nano Temper, Germany).

### Statistical analysis

To evaluate the statistical significance of the survival rate under acid stress and the pHi value, a *t* test was performed. All experiments were performed in triplicate, and data were presented as the mean ± standard deviation.

## SUPPLEMENTAL MATERIAL

Supplemental Material jb.00502-25-s0001.docx Fig. S1 to S12; Tables S1 to S4.

ASM does not own the copyrights to Supplemental Material that may be linked to, or accessed through, an article. The authors have granted ASM a non-exclusive, world-wide license to publish the Supplemental Material files. Please contact the corresponding author directly for reuse.
