# Supplementary material for: The MarR family regulator RmaH underlies the trade-off between acid sensitivity and antibiotic tolerance in Lactococcus lactis
Source: J Bacteriol. 2026 May 6;208(6):e00502-25. doi: 10.1128/jb.00502-25 (PMC13277315; doi:10.1128/jb.00502-25)
Supplement: Supplemental Material — Fig. S1 to S12; Tables S1 to S4. [file jb.00502-25-s0001.docx]

**Supplemental Materials for:**

**The MarR Family Regulator RmaH Underlies the Trade-off between Acid Sensitivity and Antibiotic Tolerance in *Lactococcus lactis***


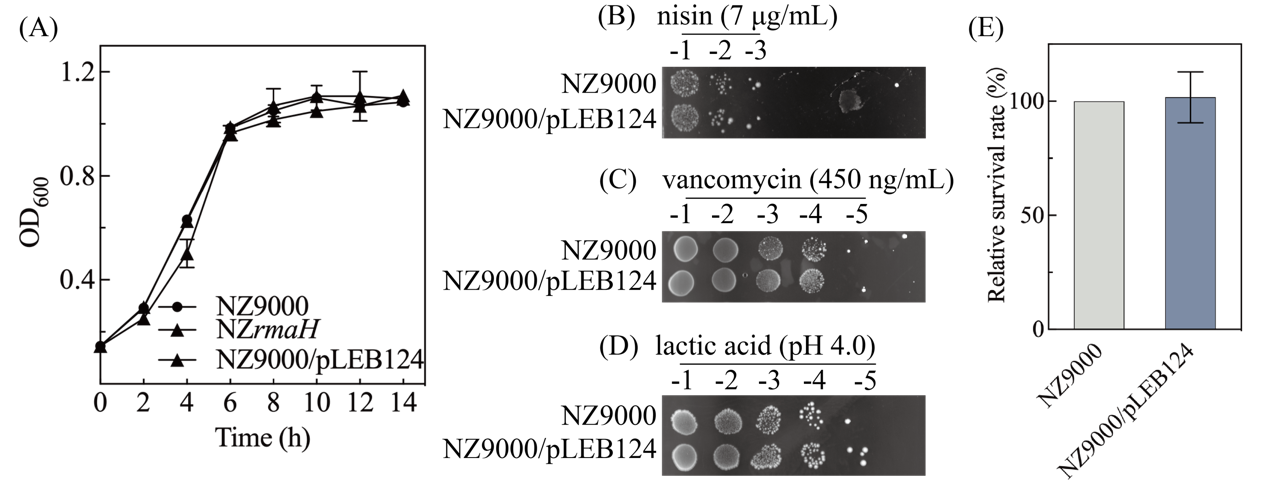


Fig. S1 Effect of the empty plasmid pLEB124 on the growth and the response of *L. lactis* to environmental stress. (A) The growth curves of *L. lactis* NZ9000, NZ9000/pLEB124 and NZ*rmaH*. (B) Nisin (7 μg/mL) tolerance test, (C) vancomycin (450 ng/mL) tolerance test, (D) lactic acid (pH 4.0, 3 h) stress test and (E) HCl (pH 3.0, 3 h) stress test (*p* < 0.001, ***).


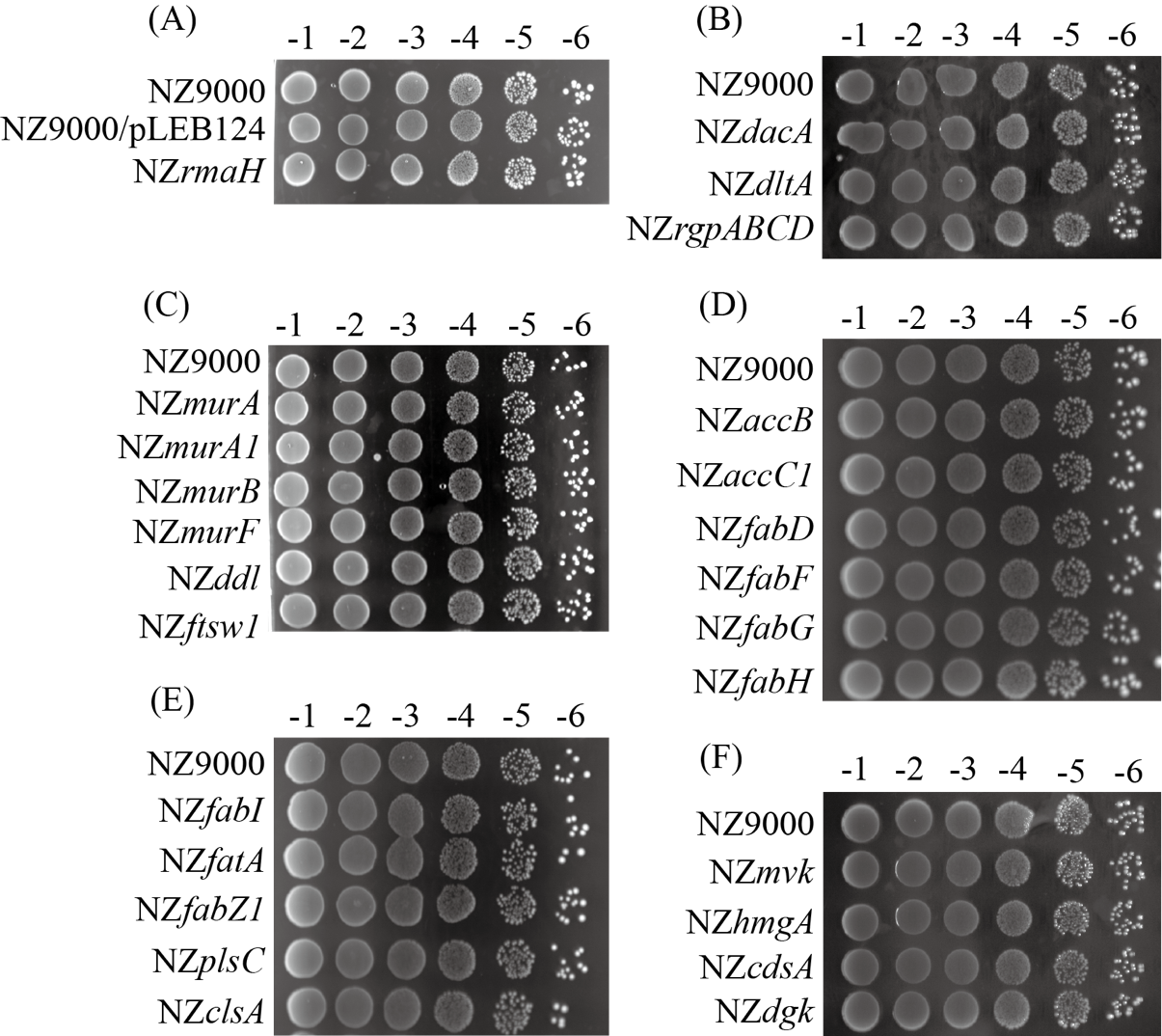


Fig. S2 Growth conditions comparison of *L. lactis* NZ9000 and the genes overexpression strains before acid stress and nisin and vancomycin tolerance assays.


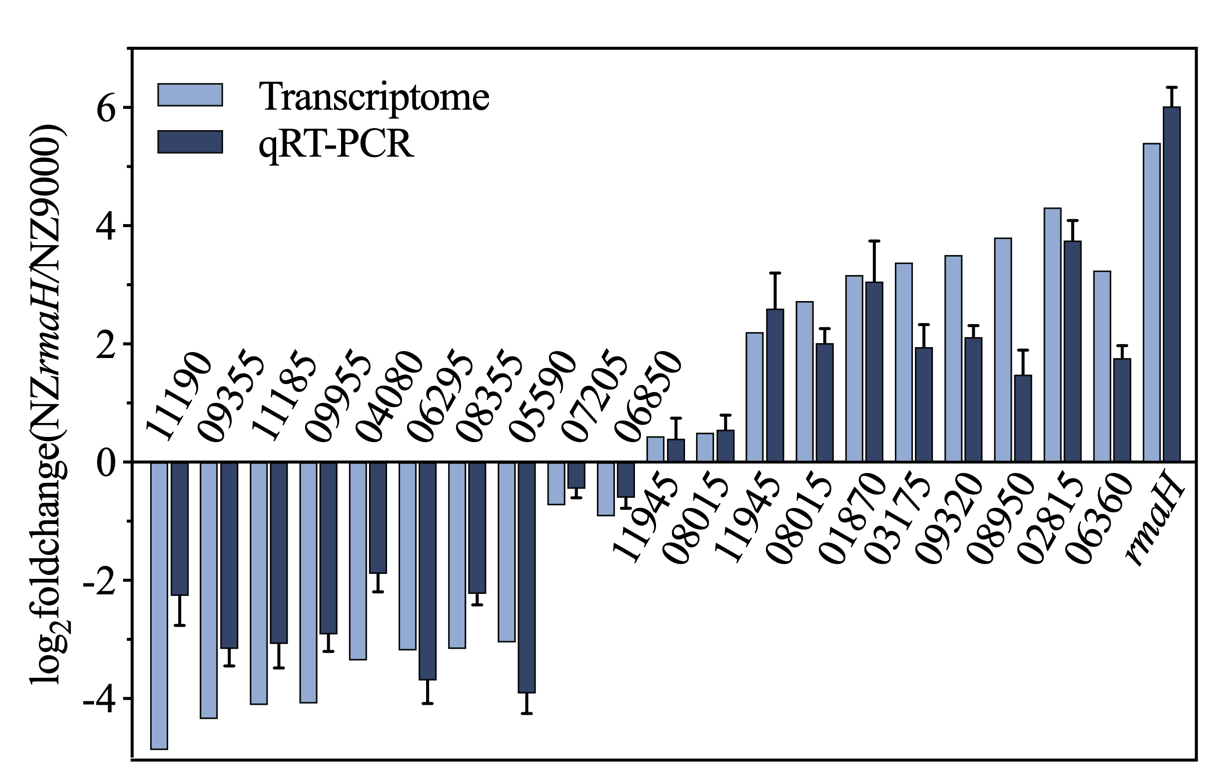


Fig. S3 Fluorescence quantification PCR result. The numbers corresponded to the gene numbers in the locus name LLNZ_XXXXX, for example, “11190” referred to “LLNZ_11190”.


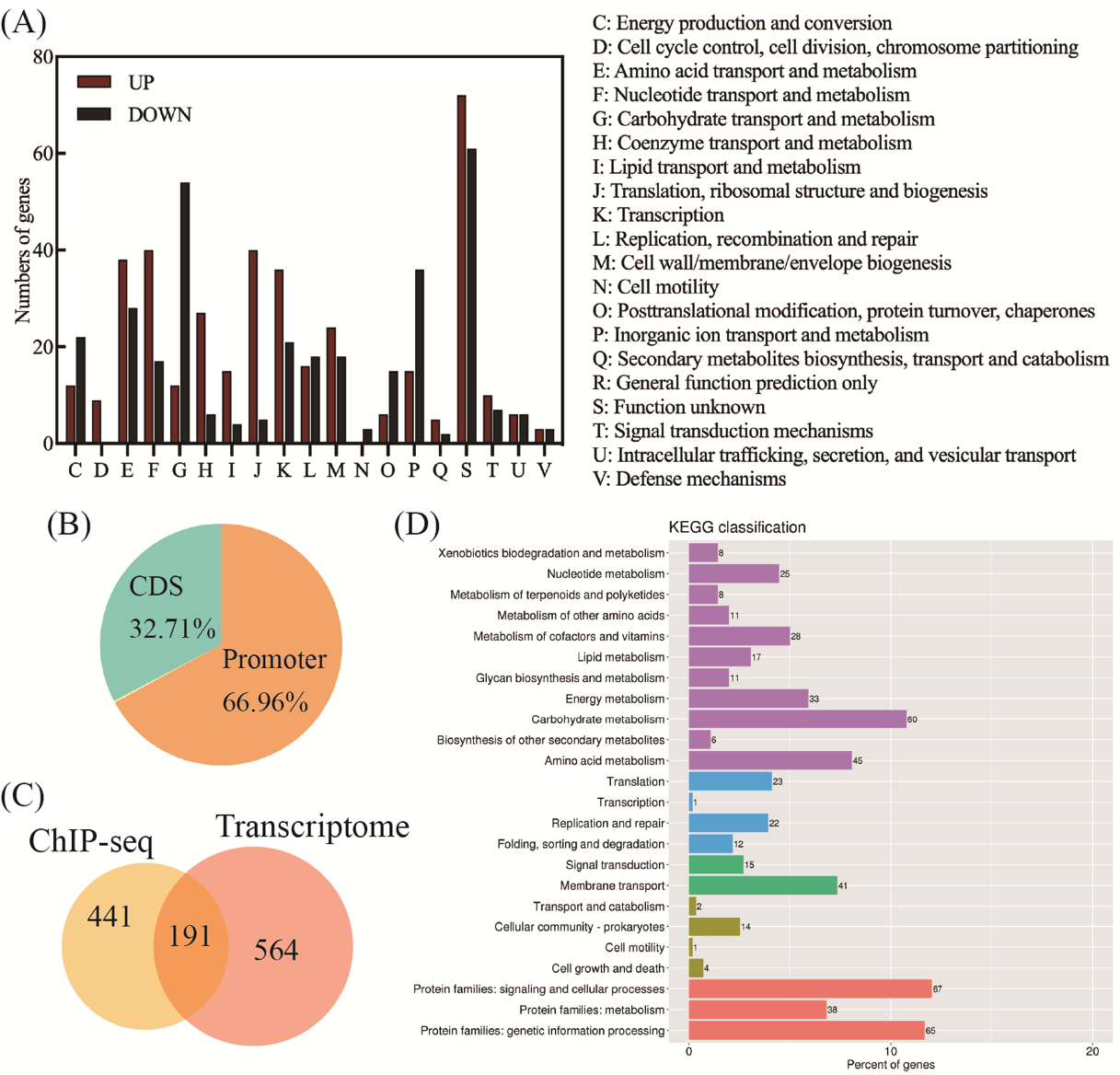


Fig. S4 The analysis of transcriptome and ChIP-seq: (A) COG function classification of significantly differentially expressed genes in NZ*rmaH*. (B) distribution of RmaH binding sites in NZ9000 genome identified in ChIP-seq, (C) comparison of transcriptome and ChIP-seq results, (D) KEGG analysis of RmaH binding genes in ChIP-seq result.


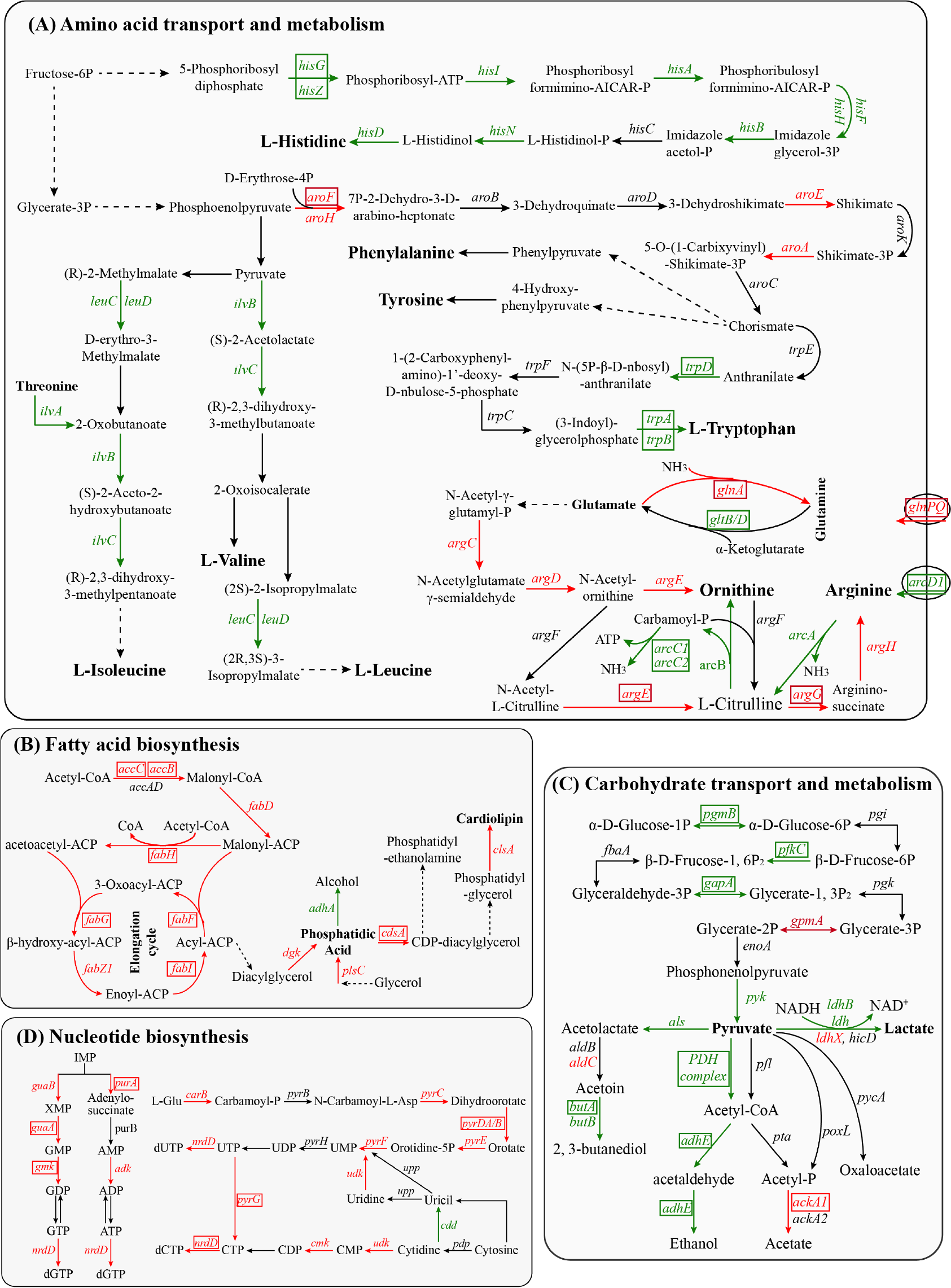


Fig. S5 Effect of RmaH on amino acid metabolism and transport (A), fatty acid biosynthesis (B), carbohydrate transport and metabolism (C) and nucleotide biosynthesis (D). Red font represented that the gene transcription level was upregulated, green font represented that the gene transcription level was downregulated and the box indicated that the gene also appeared in the ChIP-seq result.


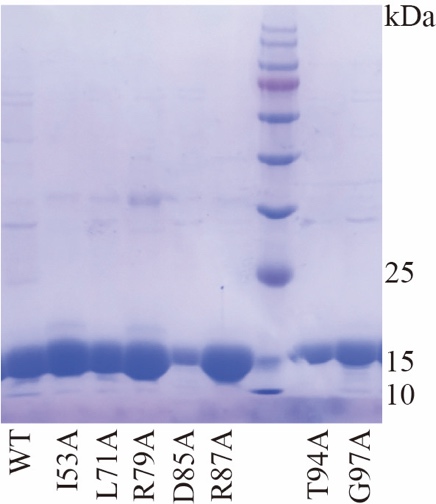


Fig. S6 The purified RmaH (WT) and its point mutation proteins analyzed by SDS-PAGE.


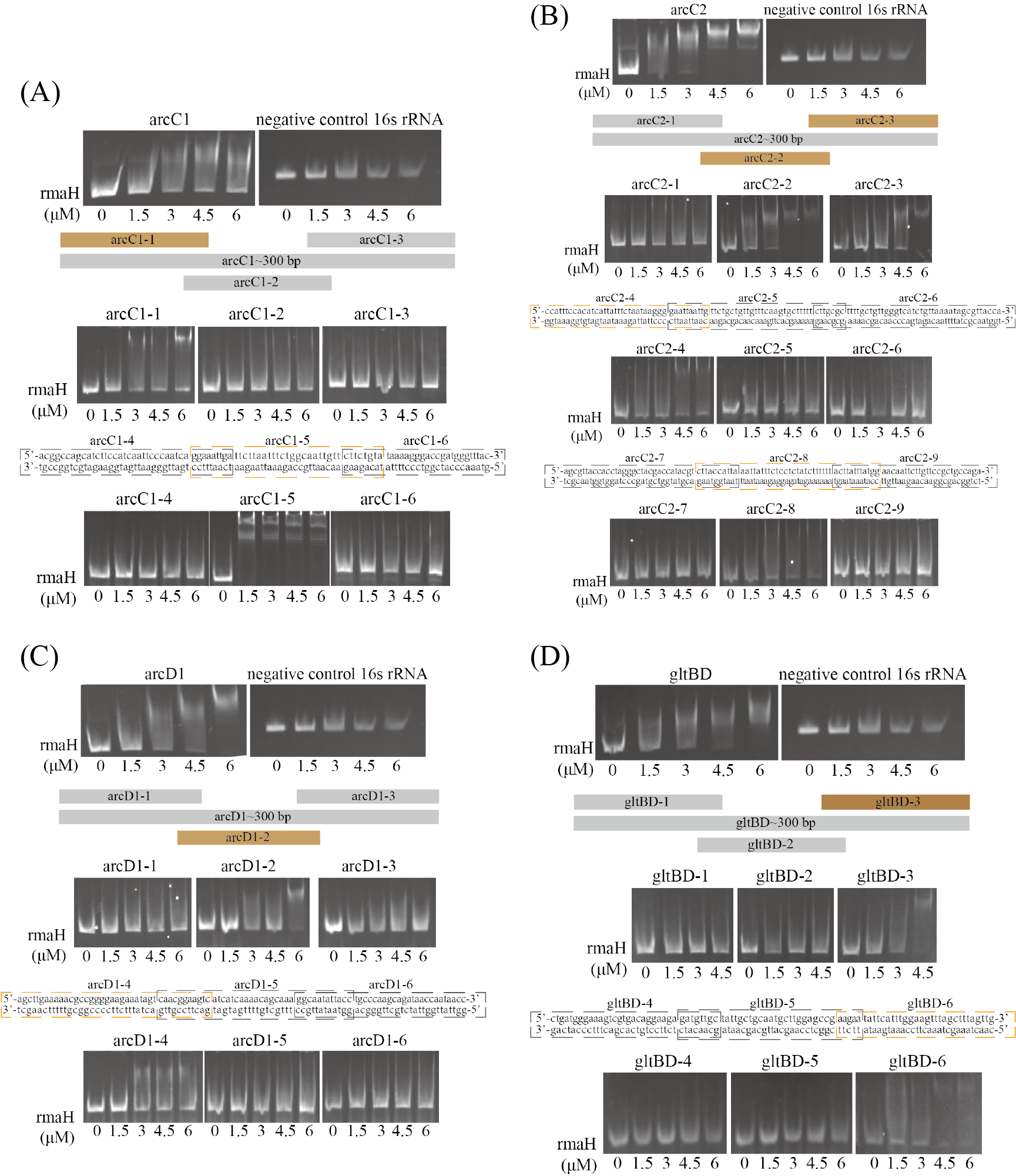


Fig. S7 Determination of the binding sites of RmaH to genes *arcC1*, *arcC2*, *arcD1* and *gltBD* by EMSA.


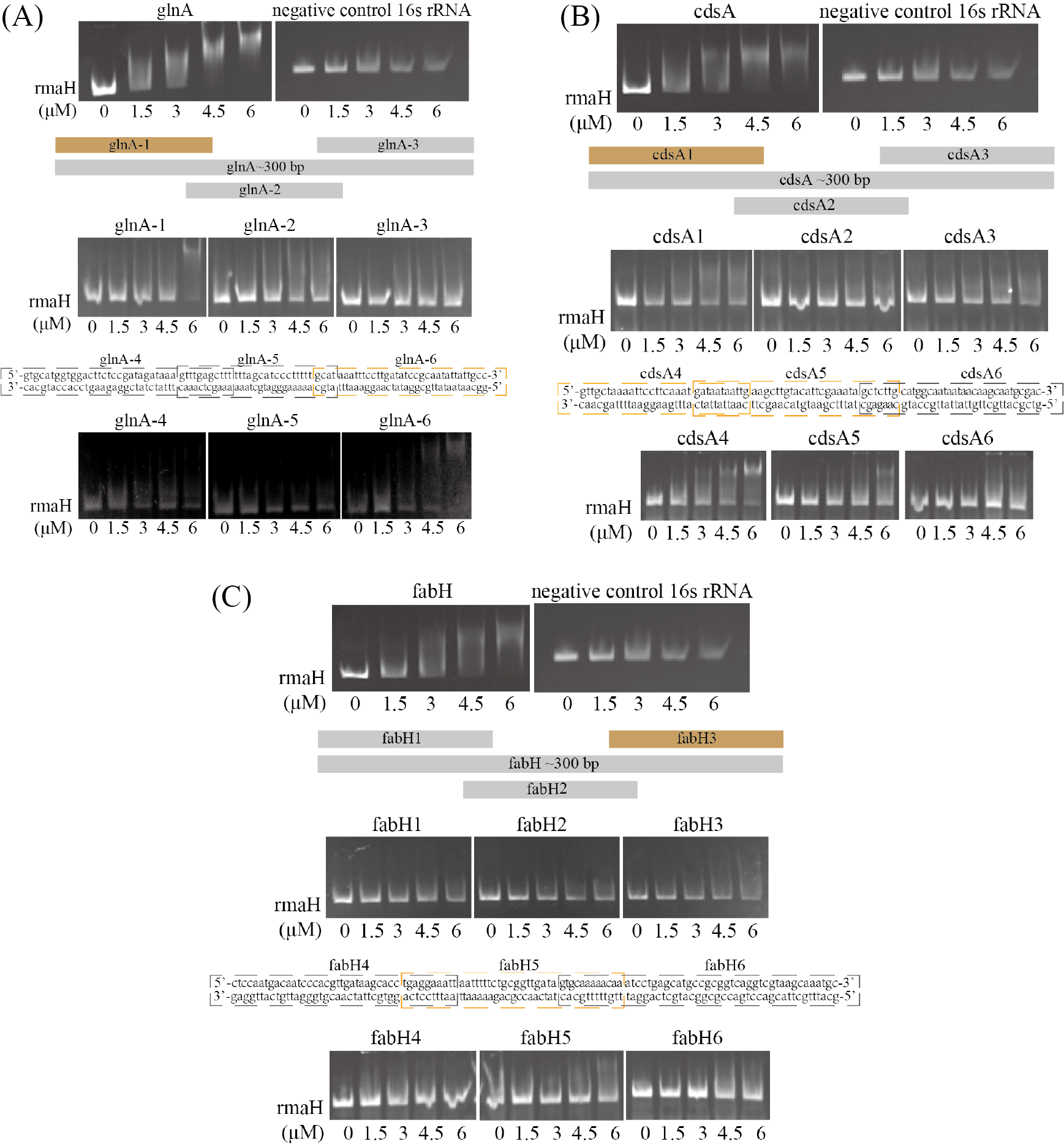


Fig. S8 Determination of the binding sites of RmaH to genes *glnA*, cds*A* and *fabH* by EMSA.


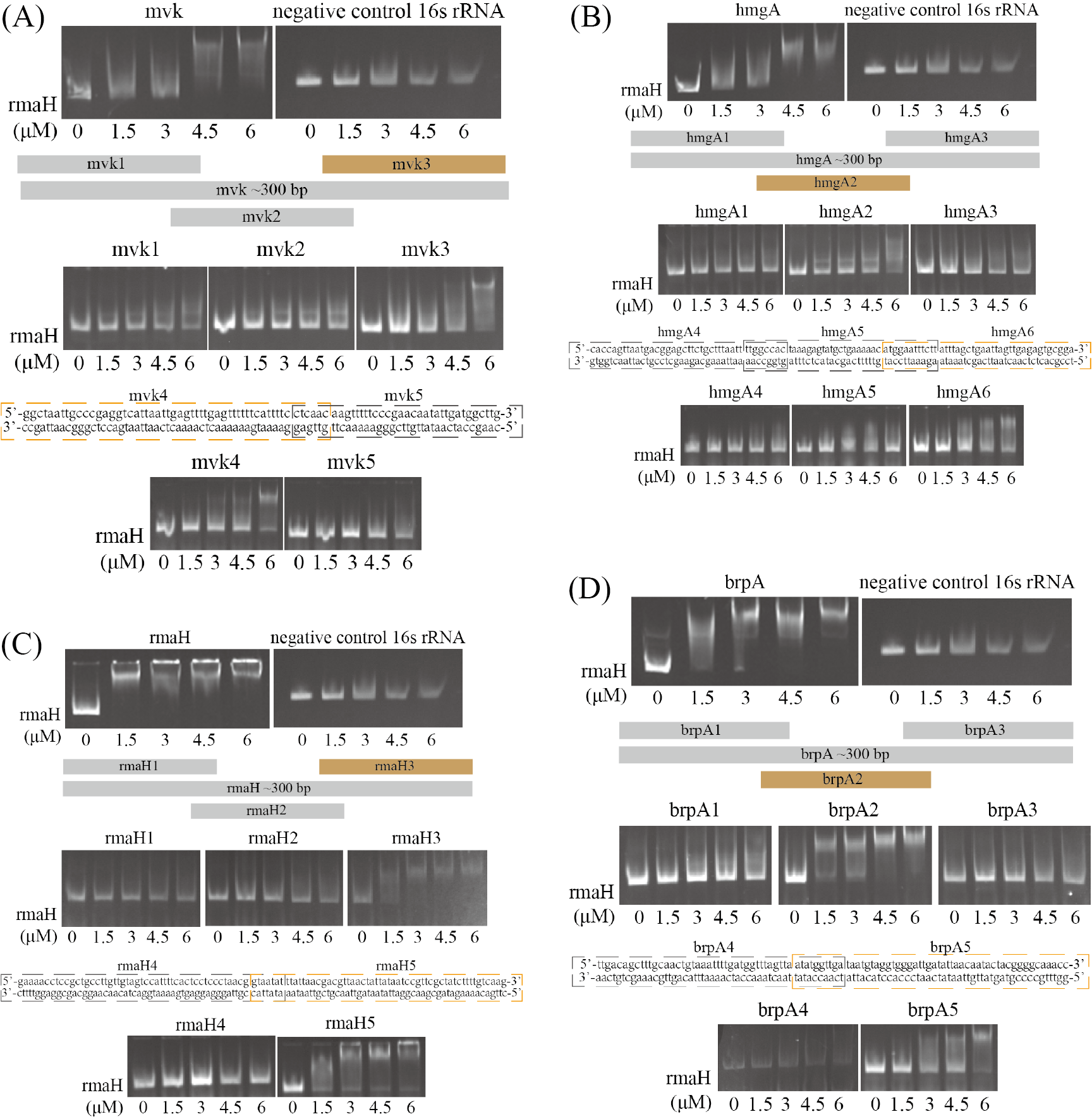


Fig. S9 Determination of the binding sites of RmaH to genes *mvk*, *hmgA*, *rmaH* and *brpA* by EMSA.


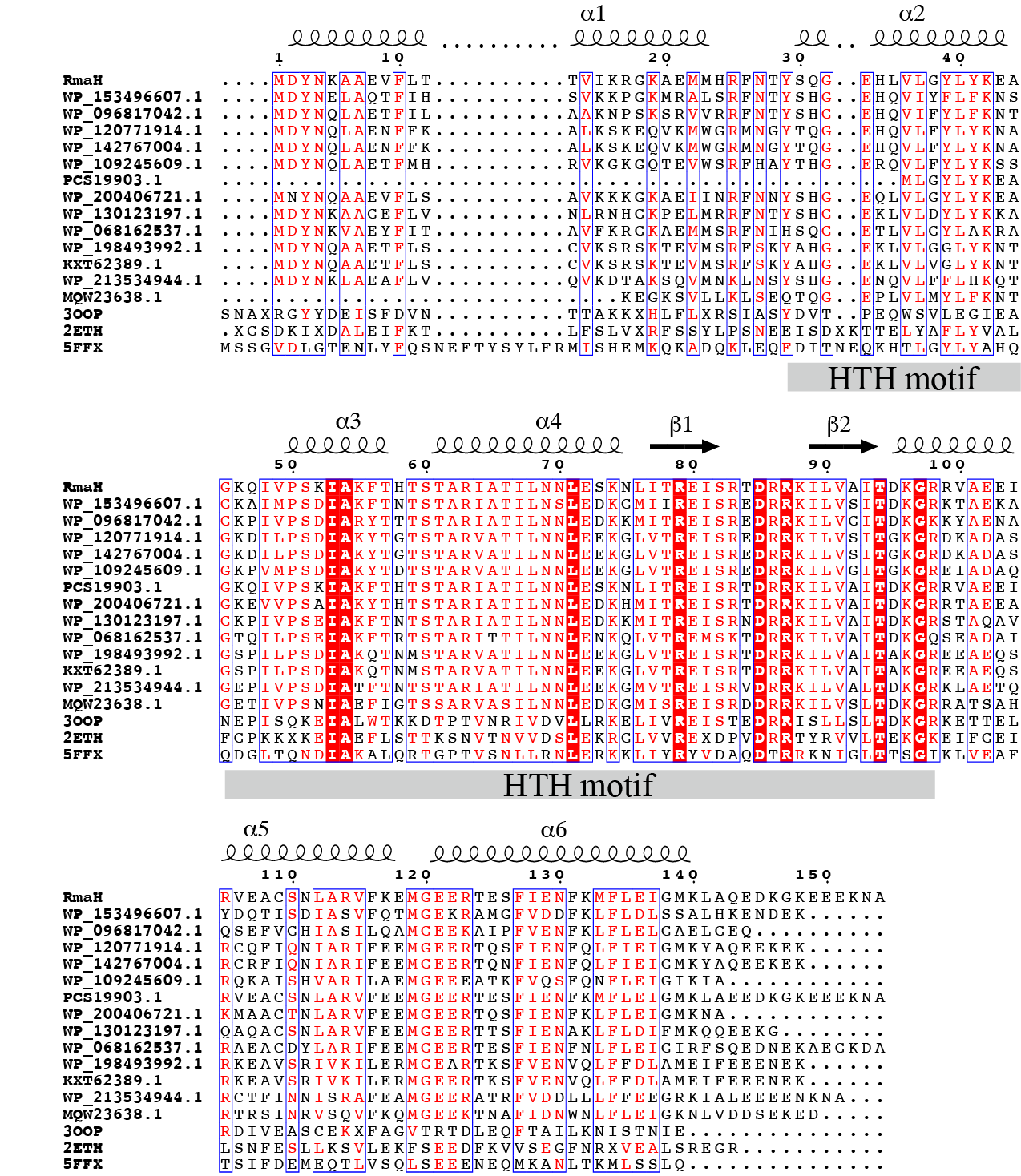


Fig. S10 Multiple sequence alignment of RmaH and its homologous proteins. The highly conserved residues (identity ≥ 70%) are in box and the residues (identity = 100%) are in red shadow. The homologous protein and its source strain were as follows: WP_153496607.1 (*L. hircilactis*), WP_096817042.1 (*L. fujiensis*), WP_120771914.1 (*L. allomyrinae*), WP_142767004.1 (*Lactococcus sp.* KACC 19320), WP_109245609.1 (*L. termiticola*), PCS19903.1 (*L. tructae*), WP_200406721.1 (*L. taiwanensis*), WP_130123197.1 (*Lactococcus sp.* S-13), WP_068162537.1 (*L. plantarum*), WP_198493992.1 (*L. garvieae*), KXT62389.1 (*Lactococcus sp.* DD01), WP_213534944.1 (*L. nasutitermitis*), MQW23638.1 (*Lactococcus sp. dk101*), 3OOP (*Listeria innocua Clip*11262), 2ETH (*Thermotoga maritima*) and 5FFX (*Staphylococcus aureus*).


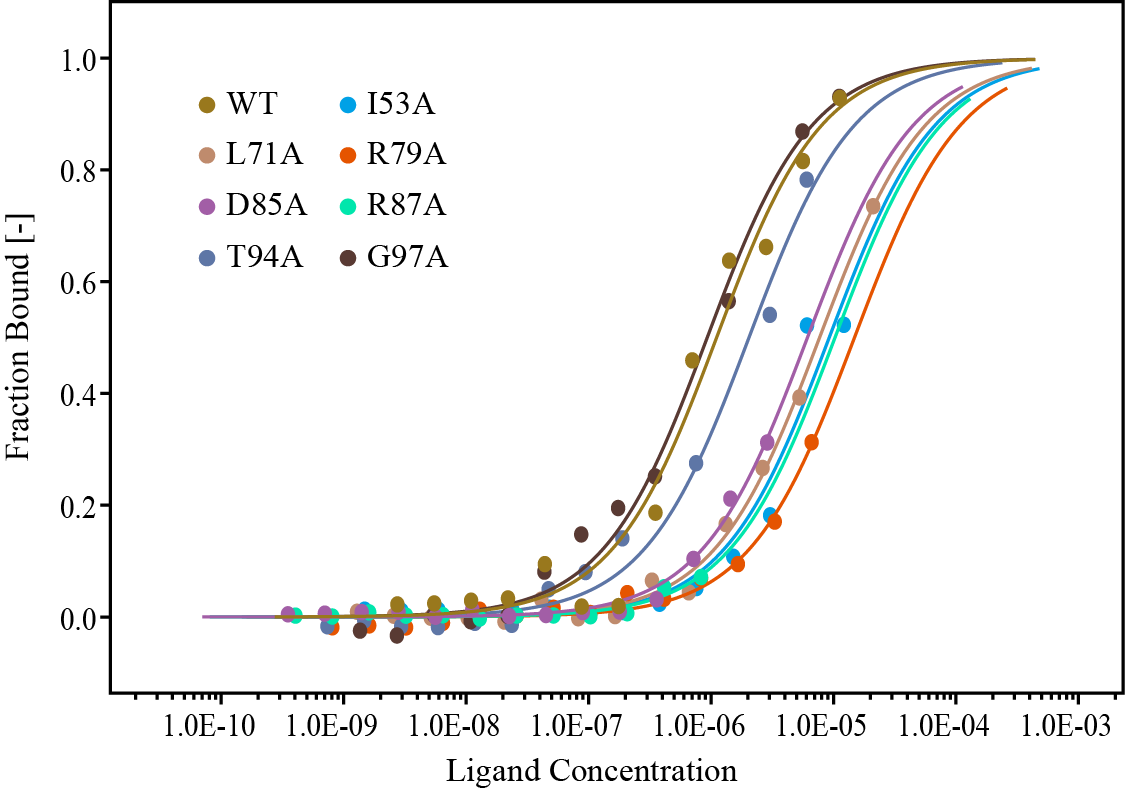


Fig. S11 Kinetic curve of the bindings of RmaH and its mutants to the DNA sequence of *rmaH*.


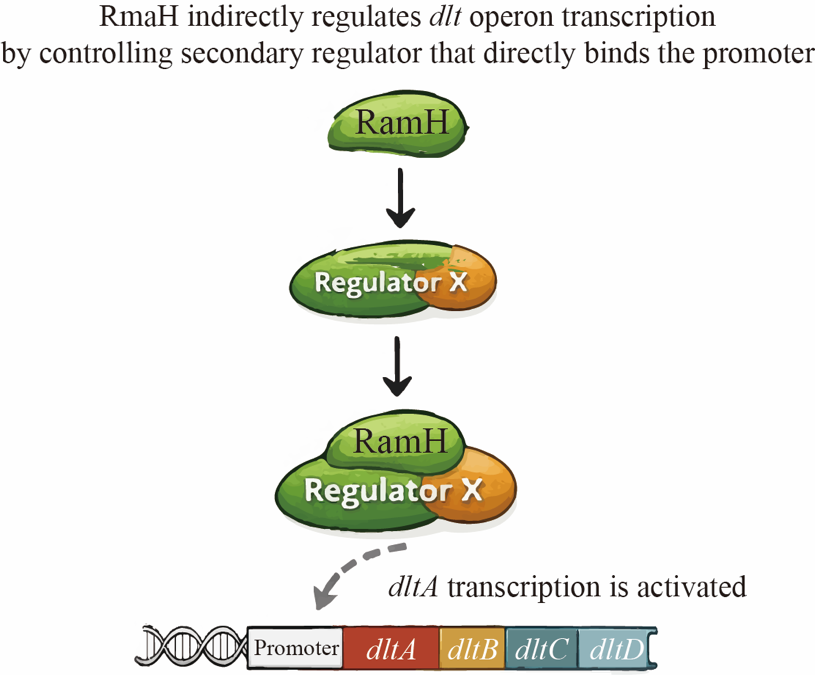


Fig. S12 Schematic diagram of RmaH indirectly regulating dlt operon transcription. RmaH indirectly regulates *dlt* operon transcription by controlling a secondary regulator that directly binds the promoter.

Table S1 Strains and plasmids used in this study

| **Strains/Plasmids** | **Characteristics** | **Source** |
| --- | --- | --- |
| **Strains** |  |  |
| *L. lactis* NZ9000 | Wild type | Laboratory stock |
| *E. coli* TG1 | Plasmid preparation | Laboratory stock |
| *E. coli* BL21 | Protein expression and purification | Laboratory stock |
| NZ9000/pLEB124 | *L. lactis* NZ9000 containing pLEB124, Em^r^ | In this study |
| NZ*rmaH*-3Flag | *L. lactis* NZ9000 containing pLEB124-*rmaH*-3Flag, Em^r^ | In this study |
| NZ*murA* | *L. lactis* NZ9000 containing pLEB124-*murA*, Em^r^ | In this study |
| NZ*murA1* | *L. lactis* NZ9000 containing pLEB124-*murA1*, Em^r^ | In this study |
| NZ*murB* | *L. lactis* NZ9000 containing pLEB124-*murB*, Em^r^ | In this study |
| NZ*murF* | *L. lactis* NZ9000 containing pLEB124-*murF*, Em^r^ | In this study |
| NZ*ddl* | *L. lactis* NZ9000 containing pLEB124-*ddl*, Em^r^ | In this study |
| NZ*ftsw1* | *L. lactis* NZ9000 containing pLEB124-*ftsw1*, Em^r^ | In this study |
| NZ*dacA* | *L. lactis* NZ9000 containing pLEB124-*dacA*, Em^r^ | In this study |
| NZ*dltA* | *L. lactis* NZ9000 containing pLEB124-*dltA*, Em^r^ | In this study |
| NZ*rgpABCD* | *L. lactis* NZ9000 containing pLEB124-*rgpABCD*, Em^r^ | In this study |
| NZ*accB* | *L. lactis* NZ9000 containing pLEB124-*accB*, Em^r^ | In this study |
| NZ*accC1* | *L. lactis* NZ9000 containing pLEB124-*accC1*, Em^r^ | In this study |
| NZ*fabD* | *L. lactis* NZ9000 containing pLEB124-*fabD*, Em^r^ | In this study |
| NZ*fabF* | *L. lactis* NZ9000 containing pLEB124-*fabF*, Em^r^ | In this study |
| NZ*fabH* | *L. lactis* NZ9000 containing pLEB124-*fabH*, Em^r^ | In this study |
| NZ*fabG* | *L. lactis* NZ9000 containing pLEB124-*fabG*, Em^r^ | In this study |
| NZ*mvk* | *L. lactis* NZ9000 containing pLEB124-*mvk*, Em^r^ | In this study |
| NZ*hmgA* | *L. lactis* NZ9000 containing pLEB124-*hmgA*, Em^r^ | In this study |
| NZ*cdsA* | *L. lactis* NZ9000 containing pLEB124-*cdsA*, Em^r^ | In this study |
| NZ*dgk* | *L. lactis* NZ9000 containing pLEB124-*dgk*, Em^r^ | In this study |
| BL21/pET28a-*rmaH* | *E. coli* BL21 containing pET28a-*rmaH*, Kana^r^ | In this study |
| BL21/RmaH(I53A) | *E. coli* BL21 containing pET28a-*rmaH*(I53A), Kana^r^ | In this study |
| BL21/RmaH(L71A) | *E. coli* BL21 containing pET28a-*rmaH*(L71A), Kana^r^ | In this study |
| BL21/RmaH(R79A) | *E. coli* BL21 containing pET28a-*rmaH*(R79A), Kana^r^ | In this study |
| BL21/RmaH(D85A) | *E. coli* BL21 containing pET28a-*rmaH*(D85A), Kana^r^ | In this study |
| BL21/RmaH(R87A) | *E. coli* BL21 containing pET28a-*rmaH*(R87A), Kana^r^ | In this study |
| BL21/RmaH(T94A) | *E. coli* BL21 containing pET28a-*rmaH*(T94A), Kana^r^ | In this study |
| BL21/RmaH(G97A) | *E. coli* BL21 containing pET28a-*rmaH*(G97A), Kana^r^ | In this study |
| **Plasmids** |  |  |
| pLEB124 | Expression vector with constitutive promoter P_45_, Em^r^ | Laboratory stock |
| pET-28a | Protein expressing vector with inducible promoter P_T7_, Kana^r^ | Laboratory stock |
| pLEB124-*rmaH*-3Flag | pLEB124 carrying genes *rmaH* with 3Flag-tag, Em^r^ | In this study |
| pLEB124-*murA* | pLEB124 carrying gene *icaR*, Em^r^ | In this study |
| pLEB124-*murA1* | pLEB124 carrying gene *murA1*, Em^r^ | In this study |
| pLEB124-*murB* | pLEB124 carrying gene *murB*, Em^r^ | In this study |
| pLEB124-*murF* | pLEB124 carrying gene *murF*, Em^r^ | In this study |
| pLEB124-*ddl* | pLEB124 carrying gene *ddl*, Em^r^ | In this study |
| pLEB124-*ftsw1* | pLEB124 carrying gene *ftsw1*, Em^r^ | In this study |
| pLEB124-*dacA* | pLEB124 carrying gene *dacA*, Em^r^ | In this study |
| pLEB124-*dltA* | pLEB124 carrying gene *dltA*, Em^r^ | In this study |
| pLEB124-*rgpABCD* | pLEB124 carrying genes *rgpABCD*, Em^r^ | In this study |
| pLEB124-*accB* | pLEB124 carrying gene *accB*, Em^r^ | In this study |
| pLEB124-*accC1* | pLEB124 carrying gene *accC1*, Em^r^ | In this study |
| pLEB124-*fabD* | pLEB124 carrying gene *fabD*, Em^r^ | In this study |
| pLEB124-*fabF* | pLEB124 carrying gene *fabF*, Em^r^ | In this study |
| pLEB124-*fabG* | pLEB124 carrying gene *fabG*, Em^r^ | In this study |
| pLEB124-*fabH* | pLEB124 carrying gene *fabH*, Em^r^ | In this study |
| pLEB124-*mvk* | pLEB124 carrying gene *mvk*, Em^r^ | In this study |
| pLEB124-*hmgA* | pLEB124 carrying genes *hmgA*, Em^r^ | In this study |
| pLEB124-*cdsA* | pLEB124 carrying gene *cdsA*, Em^r^ | In this study |
| pLEB124-*dgk* | pLEB124 carrying gene *dgk*, Em^r^ | In this study |
| pET28a-*rmaH*(I53A) | pET28a carrying gene *rmaH* that the amino acid residue I53 was mutated to A, Kana^r^ | In this study |
| pET28a-*rmaH*(L71A) | pET28a carrying gene *rmaH* that the amino acid residue L71 was mutated to A, Kana^r^ | In this study |
| pET28a-*rmaH*(R79A) | pET28a carrying gene *rmaH* that the amino acid residue R79 was mutated to A, Kana^r^ | In this study |
| pET28a-*rmaH*(D85A) | pET28a carrying gene *rmaH* that the amino acid residue D85 was mutated to A, Kana^r^ | In this study |
| pET28a-*rmaH*(R87A) | pET28a carrying gene *rmaH* that the amino acid residue R87 was mutated to A, Kana^r^ | In this study |
| pET28a-*rmaH*(T94A) | pET28a carrying gene *rmaH* that the amino acid residue T94 was mutated to A, Kana^r^ | In this study |
| pET28a-*rmaH*(G97A) | pET28a carrying gene *rmaH* that the amino acid residue G97 was mutated to A, Kana^r^ | In this study |

Table S2 Primers used in this study

| **Primers** | **Sequences (5’-3’)** | **Function** |
| --- | --- | --- |
| rmaH-3Flag-124F | CGGGATCCGATGGACTACAACAAGGCAGC（BamHI） | To construct the *rmaH* expression plasmid with 3Flag tag |
| rmaH-3Flag-124R | TCCCCCGGGCTATTTATCGTCGTCATCTTTGTAGTCGATATCATGATCTTTATAATCACCGTCATGGTCTTTGTAGTCTTCCTCCTCTTTACCTTTATCTTC（SmaI） |  |
| murA-124F | GTATTACTGAAGGGAACCTAGAATAGTGAAACTTCAAGGAGAAGTCG | To construct the overexpression plasmid (pLEB124 derived) of corresponding genes |
| murA-124R | AGGAGCACGATCATGCGCACCATTATTCTTCTCCGTCTTCATC |  |
| murA1-124F | AGGAGCACGATCATGCGCACCCAAGAAACACGATTGAGTTC |  |
| murA1-124R | GTATTACTGAAGGGAACCTAGAATAGTGGGTGGGAAGCGTATCTCTG |  |
| murB-124F | GTATTACTGAAGGGAACCTAGAATAGTGATGATAAAAGAAACTGATCTTG |  |
| murB-124R | AGGAGCACGATCATGCGCACTTAACCAATAATTCTGACCTCAGGC |  |
| murF-124F | GTATTACTGAAGGGAACCTAGAATAGTGCTCACAATTCACGAAATCGC |  |
| murF-124R | AGGAGCACGATCATGCGCACAAGTACCGACTCTTGTCAG |  |
| ddl-124F | GTATTACTGAAGGGAACCTAGAATAGTGTTGTATGGTGGTCGTTCTGCTG |  |
| ddl-124R | AGGAGCACGATCATGCGCACCACAGCCCATAAGCAGTGG |  |
| ftsw1-124F | GTATTACTGAAGGGAACCTAGAATAGTGCTTGCAGGGATTGGGATAG |  |
| ftsw1-124R | AGGAGCACGATCATGCGCACTCATGGGACACTGCTACTATAGTG |  |
| dacA-124F | AGGAGCACGATCATGCGCACAAGCTGTCAGTAAATAGACAG |  |
| dacA-124R | GTATTACTGAAGGGAACCTAGAATAGTGCTGATGAGTCTATCAGTCC |  |
| dltA-124F | AGGAGCACGATCATGCGCACCACTAGTTGTCACCTCGCTC |  |
| dltA-124R | CTGAAGGGAACCTAGAATAGTGCCTGACCTTATCGTGCTTG |  |
| rgpABCD-124F | AGGAGCACGATCATGCGCACTTACCTCAAGAGTAAGCCACTTG |  |
| rgpABCD-124R | GTATTACTGAAGGGAACCTAGAATAGTGATCATTGGGAGTCGTGGC |  |
| accB-124F | AGGAGCACGATCATGCGCACACTGACAGAGCTTTGTCAG |  |
| accB-124R | GTATTACTGAAGGGAACCTAGAATAGTGATGAACATTTCAGAAGTAAAAGA |  |
| accC1-124F | AGGAGCACGATCATGCGCACGCCATCTGACAATTCTGTCAC |  |
| accC1-124R | GTATTACTGAAGGGAACCTAGAATAGTGTTGATTGCCAATCGTGGCG |  |
| fabD-124F | AGGAGCACGATCATGCGCACGAAGGACGAACTCAATTCC |  |
| fabD-124R | GTATTACTGAAGGGAACCTAGAATAGTGTCGGGTCAGGGCGCACAAC |  |
| fabF-124F | AGGAGCACGATCATGCGCACTTACTCTCCAGTCCATTTCTTG |  |
| fabF-124R | GTATTACTGAAGGGAACCTAGAATAGTGGTTATCACTGGTTATGGTGTAG |  |
| fabH-124F | AGGAGCACGATCATGCGCACAGAGTTAAGTCAGCTTCAAC |  |
| fabH-124R | GTATTACTGAAGGGAACCTAGAATAGTGGCGAAAATCACGCAAGCG |  |
| fabG-124F | AGGAGCACGATCATGCGCACTTACATTGAAACTCCACCATC |  |
| fabG-124R | GTATTACTGAAGGGAACCTAGAATAGTGGTCTTTGTAACTGGTTCAAC |  |
| mvk-124F | AGGAGCACGATCATGCGCACAAGATGGCGATTAACCATC |  |
| mvk-124R | GTATTACTGAAGGGAACCTAGAATAGTGATGACTATCAATAAAATGGGGAC |  |
| hmgA-124F | AGGAGCACGATCATGCGCACGAACCCATACGAGTCAAACG |  |
| hmgA-124R | GTATTACTGAAGGGAACCTAGAATAGTGCAAATGTCGCCACAAGAGCG |  |
| cdsA-124F | AGGAGCACGATCATGCGCACGATTAGAACAGTCCAAGTAGATG |  |
| cdsA-124R | GTATTACTGAAGGGAACCTAGAATAGTGATGATGCAACGAATTATCACAGG |  |
| dgk-124F | AGGAGCACGATCATGCGCACCTGTCAGTAAACAATCATTCG |  |
| dgk-124R | GTATTACTGAAGGGAACCTAGAATAGTGCTCCTTGCAAATCCCAATTC |  |
| pLEB124-F | ACTGAAGGGAACCTAGAATAGTG | To confirm the correct clones of derived pLEB124 |
| pLEB124-R | TTCATTCTGCTAACCAGTAAGGC |  |
| EMSA-glnAF | AGCCAGTGGCGATAAGGTGCATGGTGGACTTCTC | EMSA |
| EMSA-glnAR | AGCCAGTGGCGATAAGAACTCACTGACTTAAGCGC |  |
| EMSA-glnPF | AGCCAGTGGCGATAAGACTTCCATAACCCCAATCG |  |
| EMSA-glnPR | AGCCAGTGGCGATAAGCGTGATTTCGTTGAACTCTG |  |
| EMSA-gltBDF | AGCCAGTGGCGATAAGGGCGCTGACAAGGTTGTC |  |
| EMSA-gltBDR | AGCCAGTGGCGATAAGGTCATGATACAACCAATCGC |  |
| EMSA-argEF | AGCCAGTGGCGATAAGACTGTAGTTGCCGGGAA |  |
| EMSA-argER | AGCCAGTGGCGATAAGCGAGCAAAATCTTCCGAAC |  |
| EMSA-argGF | AGCCAGTGGCGATAAGCCTTTAGTATCAGCATTGAG |  |
| EMSA-argGR | AGCCAGTGGCGATAAGCAGCAGGAATCGGAACAC |  |
| EMSA-arcC1F | AGCCAGTGGCGATAAGACGGCCAGCATCTTCCATC |  |
| EMSA-arcC1R | AGCCAGTGGCGATAAGAACCCAGCTATGCCATTG |  |
| EMSA-arcC2F | AGCCAGTGGCGATAAGCTGTATCAATTGGCATAGC |  |
| EMSA-arcC2R | AGCCAGTGGCGATAAGTCTGGCAGCGGAACAAG |  |
| EMSA-arcD1F | AGCCAGTGGCGATAAGCAACACCTCGCATAACAAG |  |
| EMSA-arcD1R | AGCCAGTGGCGATAAGCCTGAACTTTCAGGTGTGTC |  |
| EMSA-pgmBF | AGCCAGTGGCGATAAGGATTTGCGTTCTAATCATATCAA |  |
| EMSA-pgmBR | AGCCAGTGGCGATAAGCTCTGGTCGCCCAACTC |  |
| EMSA-pfkCF | AGCCAGTGGCGATAAGGCTAGAAAACCCTTGCAAA |  |
| EMSA-pfkCR | AGCCAGTGGCGATAAGCGTAAACTTCGATACCTTCAG |  |
| EMSA-gapAF | AGCCAGTGGCGATAAGCTTGATGGAAGTGAAACAG |  |
| EMSA-gapAR | AGCCAGTGGCGATAAGGTTGAGCATGTCCTTTCATC |  |
| EMSA-butAF | AGCCAGTGGCGATAAGGTGTCATTCCACGAACAG |  |
| EMSA-butAR | AGCCAGTGGCGATAAGCCGCTTTAGAAGCTGTTG |  |
| EMSA-pdhBF | AGCCAGTGGCGATAAGCAGCTACTGCCCAAGCAC |  |
| EMSA-pdhBR | AGCCAGTGGCGATAAGAAGATGCTGTAGCCTTCG |  |
| EMSA-pdhCF | AGCCAGTGGCGATAAGGAGCTGATTCTGGAGCCAC |  |
| EMSA-pdhCR | AGCCAGTGGCGATAAGCAATGGAAATGGTGAATCTAC |  |
| EMSA-pdhDF | AGCCAGTGGCGATAAGCATTGAAATGGCACGACC |  |
| EMSA-pdhDR | AGCCAGTGGCGATAAGGACTGACCGTGGTTTGATTG |  |
| EMSA-adhEF | AGCCAGTGGCGATAAGCGATCGTGCTGTTGAAGATC |  |
| EMSA-adhER | AGCCAGTGGCGATAAGAACACCAGCTTGTTCAGC |  |
| EMSA-adhAF | AGCCAGTGGCGATAAGGATGACTTGCCAATCTCCTG |  |
| EMSA-adhAR | AGCCAGTGGCGATAAGGTTGGAGATCGCGTGTCAG |  |
| EMSA-ackA1F | AGCCAGTGGCGATAAGCATTGATGTATCAACAGATTCTC |  |
| EMSA-ackA1R | AGCCAGTGGCGATAAGCACAATCCAGCAAATGCTGC |  |
| EMSA-fabHF | AGCCAGTGGCGATAAGGGCGTTTGAATATCAGCG |  |
| EMSA-fabHR | AGCCAGTGGCGATAAGGCATTTGCTTACGACCTGAC |  |
| EMSA-cdsAF | AGCCAGTGGCGATAAGGTTGCTAAAATTCCTTCAAATG |  |
| EMSA-cdsAR | AGCCAGTGGCGATAAGCGACTTTGATGAAGCGGA |  |
| EMSA-hmgAF | AGCCAGTGGCGATAAGCTGTTGATGAAACTCCTCG |  |
| EMSA-hmgAR | AGCCAGTGGCGATAAGGAAAGCTGAACGCCTGAG |  |
| EMSA-mvkF | AGCCAGTGGCGATAAGCATGACACCACCAAGTCC |  |
| EMSA-mvkR | AGCCAGTGGCGATAAGCAAGCCATCAATATTGTTCG |  |
| EMSA-rgpCF | AGCCAGTGGCGATAAGACTCGGGATTACAATCTGG |  |
| EMSA-rgpCR | AGCCAGTGGCGATAAGCATTGTAAAGAGGGTTAACTC |  |
| EMSA-rmaHF | AGCCAGTGGCGATAAGGTTGCTATTCTCGCTGTAC |  |
| EMSA-rmaHR | AGCCAGTGGCGATAAGGAATGCCTTTGCTCGAATTG |  |
| EMSA-brpAF | AGCCAGTGGCGATAAGCTAGGTTATAACTCAGGGAC |  |
| EMSA-brpAR | AGCCAGTGGCGATAAGGCTTGATCCCCATTAATGTG |  |
| I53A-F | GCGTGAATTTTGCAGCCTTACTTGGAACAATTTGC | To construct the point mutant protein expression plasmids (pET28a derived) |
| I53A-R | GCAAATTGTTCCAAGTAAGGCTGCAAAATTCACGC |  |
| L71A-F | CGAGTAATCAAGTTTTTACTTTCAGCATTATTTAAGATTGTTGC |  |
| L71A-R | GCAACAATCTTAAATAATGCTGAAAGTAAAAACTTGATTACTCG |  |
| R79A-F | GATCTGTTCTTGAAATCTCAGCAGTAATCAAGTTTTTACTTTC |  |
| R79A-R | GAAAGTAAAAACTTGATTACTGCTGAGATTTCAAGAACAGATC |  |
| D85A-F | GCAACCAAAATTTTTCGACGAGCTGTTCTTGAAATCTCACG |  |
| D85A-R | CGTGAGATTTCAAGAACAGCTCGTCGAAAAATTTTGGTTGC |  |
| R87A-F | CAGTAATTGCAACCAAAATTTTTGCACGATCTGTTCTTGA |  |
| R87A-R | TCAAGAACAGATCGTGCAAAAATTTTGGTTGCAATTACTG |  |
| T94A-F | GTCGACCTTTGTCAGCAATTGCAACCAAAATTTTTCG |  |
| T94A-R | CGAAAAATTTTGGTTGCAATTGCTGACAAAGGTCGAC |  |
| G97A-F | CTTCTGCGACACGTCGAGCTTTGTCAGTAATTGCAAC |  |
| G97A-R | GTTGCAATTACTGACAAAGCTCGACGTGTCGCAGAAG |  |

Table S3 Significantly differentially expressed genes in NZ*rmaH*

| **Gene ID** | **Gene name** | **Log_2_fold**  **change**  **(NZ*rmaH*/**  **NZ9000)^a^** | ***p*value** | **COG** | **Description** |
| --- | --- | --- | --- | --- | --- |
| LLNZ_RS08190 | *rmaH* | 5.40 | 4.28E-08 | COG1846 | MarR family regulator |
| **Amino acid transport and metabolism** | | | | | |
| LLNZ_RS06550 | *hisA* | -1.89 | 1.09E-08 | COG0106 | phosphoribosylformimino-5-aminoimidazole carboxamide ribotide isomerase |
| LLNZ_RS06565 | *hisB* | -1.42 | 9.40E-03 | COG0131 | imidazoleglycerol-phosphate dehydratase |
| LLNZ_RS06570 | *hisD* | -1.54 | 6.53E-08 | COG0141 | histidinol dehydrogenase |
| LLNZ_RS06545 | *hisF* | -2.75 | 7.55E-26 | COG0107 | Imidazole glycerol phosphate synthase subunit |
| LLNZ_RS06575 | *hisG* | -1.51 | 2.45E-05 | COG0040 | ATP phosphoribosyltransferase |
| LLNZ_RS06540 | *hisI* | -2.74 | 8.60E-18 | COG0140 | N5-carboxyethyl-ornithine synthase |
| LLNZ_RS06555 | *hisH* | -2.18 | 1.08E-11 | COG0118 | Imidazole glycerol phosphate synthase subunit |
| LLNZ_RS11745 | *hisN* | -4.41 | 7.15E-19 | COG0241 | Histidinol phosphatase |
| LLNZ_RS06585 | *hisZ* | -1.59 | 1.05E-05 | COG3705 | ATP phosphoribosyltransferase regulatory subunit |
| LLNZ_RS09660 | *aroC* | -1.39 | 3.82E-04 | COG0082 | chorismate synthase |
| LLNZ_RS05295 | *trpB* | -1.73 | 3.72E-09 | COG0133 | tryptophan synthase subunit beta |
| LLNZ_RS05300 | *trpA* | -1.67 | 1.30E-04 | COG0159 | tryptophan synthase alpha chain |
| LLNZ_RS05260 | *trpD* | -1.30 | 1.45E-03 | COG0547 | Anthranilate phosphoribosyltransferase |
| LLNZ_RS06475 | *ilvA* | -1.78 | 3.49E-12 | COG1171 | threonine dehydratase |
| LLNZ_RS06485 | *ilvN* | -1.77 | 4.84E-05 | COG0440 | acetolactate synthase small subunit |
| LLNZ_RS06490 | *ilvB* | -1.49 | 3.31E-03 | COG0028 | acetolactate synthase large subunit |
| LLNZ_RS06635 | *als* | -1.82 | 1.01E-04 | COG0028 | acetolactate synthase |
| LLNZ_RS06480 | *ilvC* | -1.52 | 2.08E-04 | COG0059 | Ketol-acid reductoisomerase |
| LLNZ_RS06505 | *leuD* | -1.83 | 4.75E-04 | COG0066 | 3-isopropylmalate dehydratase small subunit |
| LLNZ_RS06515 | *leuC* | -1.43 | 3.72E-06 | COG0065 | 3-isopropylmalate dehydratase large subunit |
| LLNZ_RS05995 | *gltB* | -1.41 | 1.26E-03 | COG0067 | glutamate synthase large subunit |
| LLNZ_RS05600 | *gltD* | -2.29 | 7.89E-04 | COG0493 | glutamate synthase small subunit |
| LLNZ_RS09620 | *aroA* | 1.59 | 4.51E-10 | COG0128 | 3-phosphoshikimate 1-carboxyvinyltransferase |
| LLNZ_RS09685 | *aroE* | 1.63 | 1.03E-06 | COG0169 | shikimate dehydrogenase |
| LLNZ_RS00665 | *aroF* | 1.88 | 4.62E-15 | COG0722 | Tyr-sensitive phospho-2-dehydro-deoxyheptonate aldolase |
| LLNZ_RS06205 | *aroH* | 1.42 | 1.28E-06 | COG0722 | Tyr-sensitive phospho-2-dehydro-deoxyheptonate aldolase |
| LLNZ_RS12465 | *glnA* | 1.74 | 7.71E-14 | COG0174 | glutamine synthetase |
| LLNZ_RS12470 | *glnR* | 1.74 | 2.13E-13 | COG0789 | nitrogen regulatory protein |
| LLNZ_RS02815 | *glnP* | 3.80 | 1.45E-34 | COG0834 | glutamate or arginine ABC transporter substrate binding protein |
| LLNZ_RS09895 | *glnQ* | 2.80 | 8.87E-13 | COG1126 | glutamate ABC transporter ATP-binding protein |
| LLNZ_RS08815 | *argC* | 1.64 | 3.68E-04 | COG0002 | N-acetyl-gamma-glutamyl-phosphate reductase |
| LLNZ_RS08805 | *argD* | 1.33 | 9.62E-04 | COG4992 | acetylornithine transaminase |
| LLNZ_RS02820 | *argE* | 2.18 | 2.73E-18 | COG0624 | acetylornithine deacetylase |
| LLNZ_RS00750 | *argG* | 2.57 | 4.86E-23 | COG0137 | argininosuccinate synthase |
| LLNZ_RS00755 | *argH* | 1.99 | 2.81E-18 | COG0165 | argininosuccinate lyase |
| LLNZ_RS11555 | *arcT* | -3.12 | 1.55E-34 | COG0436 | aminotransferase |
| LLNZ_RS11560 | *araC2* | -6.82 | 6.31E-128 | COG0549 | carbamate kinase |
| LLNZ_RS11565 | *araC1* | -6.87 | 6.06E-106 | COG0549 | carbamate kinase |
| LLNZ_RS11570 | *araD1* | -5.83 | 1.23E-103 | NOG03395 | arginine-ornithine antiporter |
| LLNZ_RS11575 | *arcB* | -5.62 | 3.19E-97 | COG0078 | ornithine carbamoyltransferase |
| LLNZ_RS11580 | *arcA* | -5.09 | 1.45E-83 | COG2235 | arginine deiminase |
| LLNZ_RS00630 | *ctrA* | 2.28 | 3.67E-22 | COG0531 | cationic amino acid transporter |
| LLNZ_RS01935 | *ydgB* | 1.83 | 4.12E-15 | COG1113 | amino acid permease |
| LLNZ_RS01940 | *ydgC* | 2.39 | 4.09E-24 | COG1113 | amino acid permease |
| LLNZ_RS02590 | *yfcG* | 1.65 | 3.61E-12 | COG4166 | peptide ABC transporter substrate-binding protein |
| LLNZ_RS03610 | *oppD* | 1.81 | 6.83E-15 | COG0444 | oligopeptide ABC trasporter ATP binding protein |
| LLNZ_RS04395 | *yqiA* | 1.56 | 3.32E-08 | NOG09349 | multidrug transporter |
| LLNZ_RS04520 | *--* | 1.60 | 5.93E-05 | COG1296 | branched-chain amino acid ABC transporter permease |
| LLNZ_RS06735 | *potA* | 2.15 | 1.58E-14 | COG3842 | spermidine/putrescine import ATP-binding protein |
| LLNZ_RS07330 | *ylcA* | 2.07 | 3.48E-11 | COG0531 | amino acid permease |
| LLNZ_RS08020 | *yjgC* | 3.84 | 1.55E-43 | COG0834 | amino acid ABC transporter substrate-binding protein |
| LLNZ_RS08235 | *eamA* | 1.99 | 1.05E-09 | COG0697 | EamA family transporter |
| LLNZ_RS09320 | *dtpT* | 3.38 | 2.46E-41 | COG3104 | di-/tripeptide transporter |
| LLNZ_RS09965 | *yjeM* | 2.07 | 1.06E-11 | COG0531 | glutamate/gamma-aminobutyrate family transporter |
| LLNZ_RS10045 | *ysjA* | 1.97 | 1.47E-13 | COG1113 | amino acid permease |
| LLNZ_RS12260 | *lmrP* | 1.62 | 1.01E-04 | NOG07840 | integral membrane protein |
| LLNZ_RS12430 | *lysP* | 1.81 | 2.23E-12 | COG0833 | lysine specific permease |
| LLNZ_RS12610 | *yxbD* | 3.06 | 7.83E-36 | COG0477 | MFS transporter |
| LLNZ_RS03630 | *oppA* | -2.03 | 7.53E-05 | COG0747 | oligopeptide-binding protein |
| LLNZ_RS06125 | *--* | -3.05 | 9.95E-16 | COG0477 | MFS transporter |
| LLNZ_RS10105 | *--* | -2.85 | 6.58E-05 | COG0747 | oligopeptide ABC transporter substrate-binding protein |
| LLNZ_RS10295 | *yteD* | -2.15 | 4.71E-04 | COG0477 | transmembrane efflux protein |
| LLNZ_RS04470 | *ads* | 2.12 | 5.39E-17 | COG0136 | aspartate-semialdehyde dehydrogenase |
| LLNZ_RS02940 | *glyA* | 1.81 | 2.97E-14 | COG0112 | serine hydroxymethyltransferase |
| LLNZ_RS01835 | *yddD* | 1.52 | 1.70E-07 | COG0346 | VOC family protein |
| LLNZ_RS08100 | *--* | -2.18 | 1.01E-03 | COG2755 | SGNH/GDSL hydrolase family protein |
| LLNZ_RS08260 | *butB* | -1.64 | 1.47E-11 | COG1063 | butanediol dehydrogenase |
| LLNZ_RS09395 | *pepP* | -1.94 | 3.04E-16 | COG0006 | aminopeptidase P |
| LLNZ_RS04485 | *dapA* | -1.72 | 2.52E-07 | COG0329 | dihydrodipicolinate synthase |
| **Pyruvate biogenesis and metabolism** | | | | | |
| LLNZ_RS07215 | *ldhX* | 2.58 | 5.44E-23 | COG0039 | L-lactate dehydrogenase |
| LLNZ_RS07390 | *aldC* | 4.84 | 1.16E-56 | COG3527 | Alpha-acetolactate decarboxylase |
| LLNZ_RS11470 | *ackA1* | 2.39 | 9.13E-21 | COG0282 | Acetate kinase |
| LLNZ_RS01840 | *gpmA* | 3.40 | 9.06E-44 | COG0588 | 2,3-bisphosphoglycerate-dependent phosphoglycerate mutase |
| LLNZ_RS02020 | *ldhB* | -1.65 | 2.10E-05 | COG0039 | L-lactate dehydrogenase |
| LLNZ_RS05705 | *ldhA* | -1.65 | 1.29E-02 | COG0039 | L-lactate dehydrogenase |
| LLNZ_RS06635 | *als* | -1.82 | 1.01E-04 | COG0028 | Acetolactate synthase |
| LLNZ_RS08255 | *butA* | -3.72 | 3.85E-39 | COG1028 | Acetoin reductase |
| LLNZ_RS08260 | *butB* | -1.64 | 1.47E-11 | COG1063 | 2,3-butanediol dehydrogenase |
| LLNZ_RS00405 | *pdhA* | -1.41 | 3.91E-08 | COG1071 | PDH E1 component beta subunit |
| LLNZ_RS00400 | *pdhB* | -2.38 | 4.95E-06 | COG0022 | PDH E1 component beta subunit |
| LLNZ_RS00395 | *pdhC* | -2.73 | 1.28E-06 | COG0508 | Dihydrolipoamide acetyltransferase component of PDH complex |
| LLNZ_RS00390 | *pdhD* | -3.02 | 1.48E-06 | COG1249 | Lipoamide dehydrogenase component of PDH complex |
| LLNZ_RS09985 | *pflA* | -1.70 | 7.34E-13 | COG1180 | Pyruvate-formate lyase activating enzyme |
| LLNZ_RS09955 | *adhA* | -4.08 | 3.23E-17 | COG1064 | Alcohol dehydrogenase |
| LLNZ_RS12200 | *adhE* | -1.58 | 9.55E-05 | COG1012 | Alcohol-acetaldehyde dehydrogenase |
| LLNZ_RS02325 | *pgmB* | -5.31 | 5.56E-80 | COG0637 | beta-phosphoglucomutase |
| LLNZ_RS05695 | *pfkC* | -1.68 | 3.22E-03 | COG0205 | ATP-dependent 6-phosphofructokinase |
| LLNZ_RS02795 | *gapA* | -1.61 | 3.42E-11 | COG0057 | glyceraldehyde 3-phosphate dehydrogenase |
| LLNZ_RS05700 | *pyk* | -2.02 | 1.04E-03 | COG0469 | pyruvate kinase |
| **Nucleotide transport and metabolism** | | | | | |
| LLNZ_RS01205 | *guaB* | 1.64 | 1.69E-12 | COG0517 | IMP dehydrogenase |
| LLNZ_RS05140 | *guaA* | 1.12 | 3.78E-07 | COG0518 | GMP synthase |
| LLNZ_RS10765 | *gmk* | 3.16 | 6.32E-29 | COG0194 | Guanylate kinase |
| LLNZ_RS05700 | *pyk* | -2.02 | 1.04E-03 | COG0469 | Pyruvate kinase |
| LLNZ_RS01460 | *nrdD* | 1.83 | 2.76E-14 | COG1328 | Anaerobic ribonucleoside-triphosphate reductase |
| LLNZ_RS11040 | *purA* | 3.24 | 7.22E-37 | COG0104 | Adenylosuccinate synthetase |
| LLNZ_RS04055 | *purB* | -1.14 | 9.75E-04 | COG0015 | Adenylosuccinate lyase |
| LLNZ_RS11820 | *adk* | 3.05 | 7.65E-34 | COG0563 | Adenylate kinase |
| LLNZ_RS05545 | *carB* | 1.55 | 3.28E-12 | COG0458 | Carbamoyl-phosphate synthase |
| LLNZ_RS07600 | *pyrC* | 2.81 | 1.44E-30 | COG0044 | Dihydroorotase |
| LLNZ_RS04865 | *pyrDA* | 2.13 | 1.73E-11 | COG0167 | Dihydroorotate dehydrogenase A |
| LLNZ_RS05630 | *pyrDB* | 2.31 | 3.71E-19 | COG0167 | Dihydroorotate dehydrogenase B |
| LLNZ_RS07605 | *pyrE* | 2.98 | 8.71E-26 | COG0461 | Orotate phosphoribosyltransferase |
| LLNZ_RS05635 | *pyrF* | 1.92 | 1.38E-15 | COG0284 | Orotidine-phosphate decarboxylase |
| LLNZ_RS02385 | *pyrG* | 2.08 | 2.73E-19 | COG0504 | CTP synthase |
| LLNZ_RS04565 | *pyrR* | 1.30 | 4.62E-07 | COG2065 | Pyrimidine operon regulator |
| LLNZ_RS05410 | *cdd* | -1.44 | 2.11E-03 | COG0295 | Cytidine deaminase |
| LLNZ_RS10925 | *upp* | 1.17 | 2.22E-07 | COG0035 | Uracil phosphoribosyltransferase |
| LLNZ_RS03955 | *udk* | 2.40 | 1.55E-12 | COG0572 | Uridine kinase |
| LLNZ_RS03795 | *cmk* | 2.08 | 2.10E-17 | COG0283 | Cytidylate kinase |
| **Lipid transport and metabolism** | | | | | |
| LLNZ_RS08920 | *accB* | 1.29 | 1.55E-05 | COG0511 | Biotin carboxyl carrier protein of acetyl-CoA carboxylase |
| LLNZ_RS08910 | *accC1* | 1.03 | 2.86E-06 | COG0439 | Biotin carboxylase 1 |
| LLNZ_RS02830 | *fabI* | 3.26 | 1.68E-32 | COG0623 | Enoyl-[acyl-carrier-protein] reductase |
| LLNZ_RS08945 | *fabH* | 3.23 | 4.84E-26 | COG0332 | 3-oxoacyl-[acyl-carrier-protein] synthase |
| LLNZ_RS08930 | *fabG* | 2.29 | 6.04E-22 | COG1028 | 3-oxoacyl-[acyl-carrier-protein] reductase |
| LLNZ_RS08935 | *fabD* | 2.13 | 2.63E-19 | COG0331 | Malonyl CoA-acyl carrier protein transacylase |
| LLNZ_RS02825 | *fabZ1* | 2.62 | 7.68E-15 | COG0764 | 3-hydroxyacyl-[acyl-carrier-protein] dehydratase |
| LLNZ_RS08925 | *fabF* | 1.84 | 6.48E-14 | COG0304 | 3-oxoacyl-[acyl-carrier-protein] synthase |
| LLNZ_RS07150 | *fatA* | 1.92 | 7.32E-14 | COG3884 | Oleoyl-acyl carrier protein thioesterase |
| LLNZ_RS00635 | *plsC* | 3.20 | 2.58E-28 | COG0204 | 1-acylglycerol-3-phosphate acyltransferase |
| LLNZ_RS07830 | *clsA* | 1.33 | 8.18E-08 | COG1502 | Major cardiolipin synthase |
| LLNZ_RS12095 | *uppS* | 2.97 | 3.96E-26 | COG0020 | Isoprenyl transferase |
| LLNZ_RS02180 | *mvk* | 2.55 | 1.57E-22 | COG1577 | Mevalonate kinase |
| LLNZ_RS04760 | *fadA* | 2.35 | 1.28E-21 | COG0183 | Probable acetyl-CoA acyltransferase |
| LLNZ_RS08830 | *bdh2* | 2.43 | 1.73E-21 | COG1028 | 3-hydroxybutyrate dehydrogenase |
| LLNZ_RS12090 | *cdsA* | 2.33 | 9.79E-15 | COG0575 | Phosphatidate cytidylyltransferase |
| LLNZ_RS09905 | *dgk* | 2.15 | 3.16E-11 | COG1597 | Diacylglycerol kinase |
| LLNZ_RS09155 | *menE* | 1.43 | 1.14E-09 | COG0318 | 2-succinylbenzoate--CoA ligase |
| LLNZ_RS02185 | *yeaH* | 1.43 | 1.33E-07 | COG3407 | Diphosphomevalonate decarboxylase |
| LLNZ_RS11970 | *yrhL* | -2.19 | 3.34E-06 | COG1835 | Putative peptidoglycan O-acetyltransferase |
| LLNZ_RS09810 | *vraA* | -1.76 | 1.46E-02 | COG0318 | Putative long chain fatty acid-CoA ligase |
| LLNZ_RS09955 | *adhA* | -4.08 | 3.23E-17 | COG1064 | Alcohol dehydrogenase |
| LLNZ_RS08255 | *budC* | -3.72 | 3.85E-39 | COG1028 | Diacetyl reductase |
| **Cell envelope biogenesis and cell division** | | | | | |
| LLNZ_RS02640 | *murA* | 1.86 | 1.78E-15 | COG0766 | UDP-N-acetylglucosamine 1-carboxyvinyltransferase |
| LLNZ_RS01695 | *murA1* | 2.01 | 7.49E-14 | COG0766 | UDP-N-acetylglucosamine 1-carboxyvinyltransferase |
| LLNZ_RS06740 | *murB* | 1.63 | 6.74E-11 | COG0812 | UDP-N-acetylenol-pyruvoylglucosamine reductase |
| LLNZ_RS06015 | *murI* | 2.01 | 1.65E-15 | COG0796 | glutamate racemase |
| LLNZ_RS01870 | *murF* | 2.73 | 6.40E-27 | COG0770 | UDP-N-acetylmuramoyl-tripeptide-D-alanyl-D-alanine ligase |
| LLNZ_RS01865 | *ddl* | 2.08 | 1.85E-16 | COG1181 | D-alanine-D-alanine ligase |
| LLNZ_RS03315 | *ftsw1* | 1.50 | 7.16E-10 | COG0772 | cell division protein |
| LLNZ_RS12900 | *dacA* | 1.80 | 1.76E-11 | COG1686 | D-alanyl-D-alanine carboxypeptidase |
| LLNZ_RS07395 | *murT* | 3.34 | 1.57E-27 | COG0769 | predicted UDP-N-acetylmuramyl tripeptide synthase |
| LLNZ_RS07400 | *gatD* | 2.01 | 2.32E-15 | COG3442 | glutamine amidotransferase |
| LLNZ_RS11970 | *yvhB* | -2.19 | 3.34E-06 | COG1835 | acetyltransferase |
| LLNZ_RS12095 | *uppS* | 2.97 | 3.96E-26 | COG0020 | Undecaprenyl pyrophosphate synthase |
| LLNZ_RS04470 | *asd* | 2.12 | 5.39E-17 | COG0136 | Aspartate-semialdehyde dehydrogenase |
| LLNZ_RS06165 | *dltA* | 1.54 | 1.50E-11 | COG1020 | D-alanine--poly(phosphoribitol) ligase subunit 1 |
| LLNZ_RS01115 | *rgpA* | 1.59 | 6.04E-10 | COG0438 | glycosyltransferase family 1 protein |
| LLNZ_RS01120 | *rgpB* | 1.74 | 7.42E-12 | COG0463 | α-L-Rha-α-1,3-L-rhamnosyltransferase |
| LLNZ_RS01125 | *rgpC* | 1.77 | 9.69E-10 | COG1682 | ABC transporter permease |
| LLNZ_RS01130 | *rgpD* | 1.34 | 2.03E-08 | COG1134 | ABC transporter ATP-binding protein |
| LLNZ_RS01150 | *ycbB* | 1.66 | 1.81E-11 | COG0463 | glycosyltransferase family 2 protein |
| LLNZ_RS04765 | *hmgA* | 1.58 | 1.43E-10 | COG1257 | hydroxymethylglutaryl-CoA reductase |
| LLNZ_RS08070 | *tagB* | -1.72 | 1.92E-02 | COG1887 | CDP-glycerol glycerophosphotransferase family protein |
| LLNZ_RS08085 | *tagD* | -2.20 | 1.59E-01 | COG0615 | glycerol-3-phosphate cytidylyltransferase |
| LLNZ_RS08075 | *tagF* | -1.77 | 5.58E-02 | COG1887 | CDP-Glycerol:Poly (glycerophosphate) Glycerophosphotransferase |
| LLNZ_RS10270 | *ftsA* | 1.58 | 1.05E-11 | COG0849 | cell division protein |
| LLNZ_RS07775 | *ftsE* | 2.06 | 1.55E-17 | COG2884 | cell division ATP-binding protein |
| LLNZ_RS07770 | *ftsX* | 2.12 | 4.32E-17 | COG2177 | ABC transporter permease |
| LLNZ_RS08745 | *ftsY* | 1.72 | 8.80E-12 | COG0552 | cell division protein |
| LLNZ_RS08265 | *rodA* | 2.08 | 6.89E-12 | COG0772 | rod-shape determining protein |
| LLNZ_RS00085 | *mesJ* | 1.45 | 4.10E-08 | COG0037 | cell cycle protein |
| LLNZ_RS06450 | *gidC* | 2.50 | 1.97E-21 | COG1206 | glucose-inhibited division protein |
| LLNZ_RS12590 | *mreC* | 1.22 | 1.56E-06 | COG1792 | rod shape-determining protein |
| LLNZ_RS12585 | *mreD* | 1.27 | 2.71E-07 | COG2891 | rod shape-determining protein |
| LLNZ_RS03040 | *lgt* | 2.08 | 7.50E-15 | COG0682 | prolipoprotein diacylglyceryl transferase |
| LLNZ_RS01740 | *plpA* | 4.06 | 4.10E-44 | COG1464 | MetQ/NlpA family ABC transporter substrate-binding protein |
| LLNZ_RS01745 | *plpB* | 2.91 | 7.11E-28 | COG1464 | MetQ/NlpA family ABC transporter substrate-binding protein |
| LLNZ_RS01750 | *plpC* | 1.52 | 1.25E-09 | COG1464 | MetQ/NlpA family ABC transporter substrate-binding protein |

**^a^** A positive value of log_2_foldchange(NZ*rmaH*/NZ9000) indicates that the transcription level of the gene in strain NZ*rmaH* was increased, and vice versa reduced.

Table S4 ChIP-seq result of RmaH

| **Gene ID** | **Gene name** | **Log_2_**  **ratio^a^** | **Fold_**  **Enrich-ment** | **-Log10**  **(*q*value)** | **abs_**  **summit**  **(bp)** | **COG** | **Description** |
| --- | --- | --- | --- | --- | --- | --- | --- |
| LLNZ_RS08255 | *butA* | -3.72 | 1.12 | 3.99 | 1620676 | C | acetoin reductase |
| LLNZ_RS00400 | *pdhB* | -2.38 | 1.22 | 15.67 | 69933 | C | alpha-ketoacid dehydrogenase component beta subunit |
| LLNZ_RS00395 | *pdhC* | -2.73 | 1.21 | 14.73 | 67838 | C | dihydrolipoamide acetyltransferase component of PDH complex |
| LLNZ_RS00390 | *pdhD* | -3.02 | 1.16 | 9.08 | 65641 | C | lipoamide dehydrogenase component of PDH complex |
| LLNZ_RS11470 | *ackA1* | 2.39 | 1.11 | 4.31 | 2244036 | C | Acetate kinase |
| LLNZ_RS09955 | *adhA* | -4.08 | 1.12 | 4.60 | 1979515 | C | alcohol dehydrogenase |
| LLNZ_RS12200 | *adhE* | -1.58 | 1.10 | 7.69 | 2384753 | C | alcohol-acetaldehyde dehydrogenase |
| LLNZ_RS02325 | *pgmB* | -5.31 | 1.12 | 4.33 | 452924 | C | β-phosphoglucomutase |
| LLNZ_RS02095 | *noxE* | -1.98 | 1.11 | 3.73 | 403342 | C | NADH oxidase |
| LLNZ_RS11670 | *rclA* | -2.80 | 1.14 | 12.34 | 2291559 | C | Probable pyridine nucleotide-disulfide oxidoreductase |
| LLNZ_RS09765 | *yhaP* | -1.63 | 1.15 | 6.78 | 1940008 | CP | Uncharacterized protein |
| LLNZ_RS02820 | *argE* | 2.18 | 1.18 | 9.96 | 536336 | E | acetylornithine deacetylase |
| LLNZ_RS00750 | *argG* | 2.57 | 1.22 | 14.78 | 134442 | E | Argininosuccinate synthase |
| LLNZ_RS11570 | *arcD1* | -5.83 | 1.15 | 7.56 | 2262519 | E | arginine-ornithine antiporter |
| LLNZ_RS11560 | *arcC2* | -6.82 | 1.14 | 6.22 | 2260188 | E | carbamate kinase |
| LLNZ_RS11565 | *arcC1* | -6.87 | 1.13 | 5.22 | 2260922 | E | carbamate kinase |
| LLNZ_RS12465 | *glnA* | 1.74 | 1.19 | 11.60 | 2441824 | E | glutamine synthetase |
| LLNZ_RS06000 | *gltD* | -2.29 | 1.13 | 5.07 | 1155291 | E | glutamate synthase subunit beta |
| LLNZ_RS09895 | *glnQ* | 2.80 | 1.21 | 12.56 | 1962029 | E | glutamate ABC transporter ATP-binding protein |
| LLNZ_RS06575 | *hisG* | -1.51 | 1.25 | 17.53 | 1267209 | E | ATP phosphoribosyltransferase |
| LLNZ_RS05295 | *trpB* | -1.73 | 1.16 | 7.04 | 1003447 | E | tryptophan synthase subunit beta |
| LLNZ_RS05260 | *trpD* | -1.30 | 1.22 | 13.70 | 997892 | E | Anthranilate phosphoribosyltransferase |
| LLNZ_RS00665 | *aroF* | 1.88 | 1.12 | 4.69 | 119738 | E | Tyr-sensitive phospho-2-dehydro-deoxyheptonate aldolase |
| LLNZ_RS09100 | *thrA* | 1.54 | 1.13 | 5.05 | 1801781 | E | aspartokinase/ homoserine dehydrogenase |
| LLNZ_RS09115 | *ansB* | 3.12 | 1.13 | 5.04 | 1804736 | E | asparaginase |
| LLNZ_RS04470 | *ads* | 2.12 | 1.23 | 14.56 | 841096 | E | aspartate-semialdehyde dehydrogenase |
| LLNZ_RS08690 | *sdaB* | 2.09 | 1.30 | 24.83 | 1713063 | E | beta-subunit of L-serine dehydratase |
| LLNZ_RS10185 | *cysE* | 1.57 | 1.18 | 9.64 | 2025561 | E | serine O-acetyltransferase |
| LLNZ_RS02940 | *glyA* | 1.81 | 1.18 | 9.84 | 556891 | E | serine hydroxymethyltransferase |
| LLNZ_RS00630 | *ctrA* | 2.28 | 1.29 | 26.37 | 111799 | E | cationic amino acid transporter |
| LLNZ_RS08020 | *yjgC* | 3.84 | 5.02 | 2481.72 | 1568538 | E | amino acid ABC transporter substrate-binding protein |
| LLNZ_RS09320 | *dtpT* | 3.38 | 1.25 | 18.83 | 1848396 | E | di-/tripeptide transporter |
| LLNZ_RS03400 | *bcaT* | 2.49 | 1.10 | 2.80 | 646572 | E | branched-chain amino acid transport system II carrier protein |
| LLNZ_RS09965 | *yjeM* | 2.07 | 1.10 | 2.13 | 1982605 | E | glutamate/gamma-aminobutyrate family transporter |
| LLNZ_RS10045 | *ysjA* | 1.97 | 1.25 | 19.23 | 1999539 | E | Gamma-aminobutyrate permease and related permeases |
| LLNZ_RS01935 | *ydgB* | 1.83 | 1.25 | 19.53 | 369289 | E | amino acid permease |
| LLNZ_RS12430 | *lysP* | 1.81 | 1.16 | 8.29 | 2434687 | E | lysine specific permease |
| LLNZ_RS02590 | *yfcG* | 1.65 | 1.14 | 5.91 | 500364 | E | peptide ABC transporter substrate-binding protein |
| LLNZ_RS08100 | *-* | -2.18 | 1.16 | 7.57 | 1583181 | E | SGNH/GDSL hydrolase family protein |
| LLNZ_RS10105 | *oppA* | -2.85 | 1.17 | 8.66 | 2011684 | E | oligopeptide ABC transporter substrate-binding protein |
| LLNZ_RS11040 | *purA* | 3.24 | 1.16 | 8.80 | 2169342 | F | Adenylosuccinate synthetase |
| LLNZ_RS10765 | *gmk* | 3.16 | 1.12 | 4.50 | 2118159 | F | Guanylate kinase |
| LLNZ_RS05630 | *pyrDB* | 2.31 | 1.17 | 8.45 | 1068915 | F | Dihydroorotate dehydrogenase B |
| LLNZ_RS02385 | *pyrG* | 2.08 | 1.23 | 15.68 | 462893 | F | CTP synthase |
| LLNZ_RS01460 | *nrdD* | 1.83 | 1.15 | 7.23 | 267953 | F | Anaerobic ribonucleoside-triphosphate reductase |
| LLNZ_RS05140 | *guaA* | 1.12 | 1.14 | 5.33 | 973916 | F | GMP synthase |
| LLNZ_RS03170 | *apt* | 3.45 | 1.10 | 3.41 | 596908 | F | Adenine phosphoribosyltransferase |
| LLNZ_RS11650 | *gla* | 1.57 | 1.11 | 7.36 | 2285568 | G | Glycerol facilitator-aquaporin |
| LLNZ_RS04395 | *yqiA* | 1.56 | 1.14 | 5.90 | 823755 | G | Multidrug resistance protein |
| LLNZ_RS02795 | *gapA* | -1.61 | 1.16 | 8.05 | 530974 | G | glyceraldehyde 3-phosphate dehydrogenase |
| LLNZ_RS05695 | *pfkC* | -1.68 | 3.43 | 1093.72 | 1078950 | G | ATP-dependent 6-phosphofructokinase |
| LLNZ_RS02760 | *glpT* | -1.63 | 1.36 | 36.45 | 523439 | G | Glycerol-3-phosphate transporter |
| LLNZ_RS04455 | *tkt* | -2.03 | 1.20 | 11.02 | 837672 | G | Transketolase |
| LLNZ_RS00845 | *gbeA* | -2.34 | 1.17 | 9.01 | 150252 | G | 1,4-alpha-glucan-branching enzyme |
| LLNZ_RS00140 | *mtlA* | -2.46 | 1.14 | 7.00 | 27545 | G | PTS system mannitol-specific EIICB component |
| LLNZ_RS05135 | *scrK* | -2.47 | 1.21 | 12.45 | 972840 | G | Fructokinase |
| LLNZ_RS08095 | *-* | -2.63 | 1.16 | 7.23 | 1581966 | G | - |
| LLNZ_RS02505 | *araQ* | -2.68 | 1.71 | 142.34 | 486092 | G | L-arabinose transport system permease protein |
| LLNZ_RS03895 | *ascB* | -2.82 | 1.16 | 6.92 | 739318 | G | 6-phospho-beta-glucosidase |
| LLNZ_RS02490 | *ycjT* | -2.90 | 1.22 | 14.86 | 482027 | G | Uncharacterized glycosyl hydrolase |
| LLNZ_RS04420 | *yqhA* | -3.10 | 1.13 | 4.66 | 829791 | G | aldose epimerase |
| LLNZ_RS04435 | *kdgA* | -3.39 | 1.10 | 2.94 | 833568 | G | 2-keto-3-deoxy-6-phosphogluconate aldolase |
| LLNZ_RS01005 | *bglC* | -3.49 | 1.19 | 11.81 | 178992 | G | Aryl-phospho-beta-D-glucosidase |
| LLNZ_RS04085 | *rbsB* | -4.12 | 1.19 | 10.67 | 778049 | G | Ribose import binding protein |
| LLNZ_RS03835 | *malE* | -4.80 | 1.21 | 12.56 | 723136 | G | Maltose/maltodextrin-binding protein |
| LLNZ_RS02320 | *trePP* | -4.95 | 1.23 | 16.67 | 451102 | G | Trehalose 6-phosphate phosphorylase |
| LLNZ_RS04915 | *gmuC* | -5.04 | 1.33 | 28.86 | 929918 | G | PTS system oligo-beta-mannoside-specific EIIC component |
| LLNZ_RS03845 | *dexA* | -5.62 | 1.16 | 7.94 | 725421 | G | Glucan 1,6-alpha-glucosidase |
| LLNZ_RS03855 | *-* | -6.44 | 1.24 | 16.63 | 727483 | G | Alpha-amylase |
| LLNZ_RS03860 | *malL* | -6.87 | 1.26 | 20.61 | 728844 | G | Oligo-1,6-glucosidase |
| LLNZ_RS12380 | *gntP* | -2.40 | 1.28 | 24.70 | 2425899 | GE | Gluconate permease |
| LLNZ_RS08920 | *accB* | 1.29 | 1.22 | 13.66 | 1764819 | I | Biotin carboxyl carrier protein of acetyl-CoA carboxylase |
| LLNZ_RS08910 | *accC1* | 1.03 | 1.10 | 3.11 | 1762387 | I | Biotin carboxylase 1 |
| LLNZ_RS08830 | *fabG* | 2.43 | 1.24 | 16.61 | 1747533 | IQ | 3-ketoacyl-(acyl-carrier-protein) reductase |
| LLNZ_RS08945 | *fabH* | 3.23 | 1.14 | 5.80 | 1768299 | I | 3-oxoacyl-[acyl-carrier-protein] synthase |
| LLNZ_RS02830 | *fabI* | 3.26 | 1.16 | 8.00 | 538215 | I | Enoyl-[acyl-carrier-protein] reductase |
| LLNZ_RS02180 | *mvk* | 2.55 | 1.14 | 6.50 | 416673 | I | Mevalonate kinase |
| LLNZ_RS12090 | *cdsA* | 2.33 | 1.25 | 20.42 | 2359129 | I | Phosphatidate cytidylyltransferase |
| LLNZ_RS09905 | *dgk* | 2.15 | 1.10 | 2.02 | 1965251 | I | Diacylglycerol kinase |
| LLNZ_RS04765 | *hmgA* | 1.58 | 1.10 | 2.79 | 901499 | I | hydroxymethylglutaryl-CoA reductase |
| LLNZ_RS09940 | *murJ* | 2.80 | 1.19 | 11.44 | 1976585 | M | Lipid II flippase |
| LLNZ_RS01125 | *rgpC* | 1.77 | 1.12 | 4.52 | 201963 | M | ABC transporter permease |
| LLNZ_RS03040 | *lgt* | 2.08 | 1.14 | 6.21 | 572363 | M | Prolipoprotein diacylglyceryl transferase |
| LLNZ_RS11970 | *yvhB* | -2.19 | 1.36 | 41.27 | 2335339 | M | acetyltransferase |
| LLNZ_RS05685 | *nagA* | -2.02 | 1.28 | 21.35 | 1078313 | M | N-acetylglucosamine-6-phosphate deacetylase |
| LLNZ_RS08995 | *ywbG* | 2.83 | 1.33 | 29.34 | 1778107 | M | Uncharacterized protein |
| LLNZ_RS08645 | *-* | 1.89 | 1.13 | 4.96 | 1700207 | M | - |
| LLNZ_RS09005 | *-* | -1.63 | 1.26 | 19.66 | 1780831 | M | - |
| LLNZ_RS11075 | *dus1* | 3.34 | 1.20 | 12.54 | 2175292 | J | Probable tRNA-dihydrouridine synthase |
| LLNZ_RS02360 | *truA* | 2.85 | 1.17 | 9.30 | 457965 | J | tRNA pseudouridine synthase |
| LLNZ_RS00880 | *tgt* | 2.47 | 1.13 | 5.88 | 155679 | J | Queuine tRNA-ribosyltransferase |
| LLNZ_RS12820 | *rpsI* | 2.07 | 1.20 | 13.69 | 2506684 | J | 30S ribosomal protein S9 |
| LLNZ_RS10195 | *pnp* | 1.86 | 1.10 | 3.54 | 2029014 | J | Polyribonucleotide nucleotidyltransferase |
| LLNZ_RS01905 | *prfC* | 1.71 | 1.13 | 5.73 | 359683 | J | Peptide chain release factor 3 |
| LLNZ_RS12880 | *rpsG* | 1.52 | 1.12 | 4.79 | 2517670 | J | 30S ribosomal protein S7 |
| LLNZ_RS11675 | *gltX* | -1.60 | 1.12 | 10.09 | 2292727 | J | Glutamate--tRNA ligase |
| LLNZ_RS03220 | *hpf* | -4.19 | 1.17 | 9.24 | 605384 | J | Ribosome hibernation promotion factor |
| LLNZ_RS08190 | *rmaH* | 5.41 | 2.19 | 1755.43 | 1605201 | K | MarR family transcriptional regulator |
| LLNZ_RS01570 | *rmeR* | 2.70 | 1.32 | 32.87 | 291892 | K | MerR family transcriptional regulator |
| LLNZ_RS02350 | *brpA* | 2.27 | 6.31 | 4180.90 | 456543 | K | Biofilm regulatory protein A |
| LLNZ_RS11955 | *nusG* | 2.15 | 2.80 | 747.57 | 2331849 | K | Transcription termination/antitermination protein |
| LLNZ_RS02755 | *glvR* | 1.59 | 1.18 | 10.50 | 521527 | K | HTH-type transcriptional regulator |
| LLNZ_RS08680 | *copY* | -1.51 | 1.36 | 35.92 | 1711211 | K | Transcriptional repressor |
| LLNZ_RS10660 | *-* | -1.56 | 1.40 | 46.47 | 2104156 | K | - |
| LLNZ_RS03870 | *malR* | -2.75 | 1.10 | 2.74 | 732191 | K | HTH-type transcriptional regulator |
| LLNZ_RS01910 | *cshA* | 3.61 | 1.17 | 8.96 | 362044 | L | DEAD-box ATP-dependent RNA helicase |
| LLNZ_RS01860 | *recR* | 2.96 | 1.39 | 47.52 | 346224 | L | Recombination protein |
| LLNZ_RS03165 | *recJ* | 1.94 | 1.62 | 104.70 | 595769 | L | Single-stranded-DNA-specific exonuclease |
| LLNZ_RS12250 | *ylbH* | 1.65 | 1.12 | 5.02 | 2397184 | L | Putative rRNA methyltransferase |
| LLNZ_RS00285 | *-* | -1.70 | 3.00 | 883.28 | 45947 | L | Transposase for insertion sequence element IS232 |
| LLNZ_RS04175 | *dnaC* | -1.83 | 1.18 | 9.13 | 789859 | L | DNA replication protein |
| LLNZ_RS00355 | *-* | -2.28 | 1.20 | 16.29 | 59424 | L | Transposase for insertion sequence element IS905 |
| LLNZ_RS02810 | *uvrB* | -2.66 | 1.10 | 3.17 | 533686 | L | UvrABC system protein B |
| LLNZ_RS10030 | *uvrA* | -2.68 | 1.15 | 6.64 | 1996908 | L | UvrABC system protein A |
| LLNZ_RS12500 | *mutS* | -2.98 | 1.14 | 6.38 | 2449567 | L | DNA mismatch repair protein |
| LLNZ_RS11235 | *yrrN* | 2.97 | 1.10 | 3.00 | 2208619 | O | Uncharacterized protease |
| LLNZ_RS11230 | *yrrO* | 1.74 | 1.27 | 23.50 | 2207428 | O | Uncharacterized protease |
| LLNZ_RS10455 | *clpP* | -1.66 | 1.19 | 11.16 | 2083576 | O | ATP-dependent Clp protease proteolytic subunit |
| LLNZ_RS03340 | *clpP* | -1.86 | 1.14 | 5.56 | 634617 | O | ATP-dependent Clp protease proteolytic subunit |
| LLNZ_RS01485 | *-* | -2.52 | 1.25 | 18.84 | 274941 | OS | - |
| LLNZ_RS01495 | *ecfA1* | 2.00 | 1.12 | 4.76 | 276537 | P | Energy-coupling factor transporter ATP-binding protein |
| LLNZ_RS01505 | *ecfT* | 1.75 | 1.15 | 7.26 | 277836 | P | Energy-coupling factor transporter transmembrane protein |
| LLNZ_RS12260 | *-* | 1.62 | 1.12 | 4.93 | 2399197 | P | - |
| LLNZ_RS02500 | *lacF* | -1.56 | 1.11 | 4.04 | 484144 | P | Lactose transport system permease protein |
| LLNZ_RS01630 | *phnE* | -1.62 | 1.16 | 8.09 | 300515 | P | Phosphonate transport system permease protein |
| LLNZ_RS09480 | *yqgI* | -2.14 | 1.21 | 13.85 | 1878555 | P | Probable ABC transporter permease protein |
| LLNZ_RS10115 | *oppB* | -2.32 | 1.19 | 10.87 | 2013627 | P | Oligopeptide transport system permease protein |
| LLNZ_RS12750 | *-* | -2.38 | 1.27 | 23.77 | 2495713 | P | Calcium-transporting ATPase 1 |
| LLNZ_RS05905 | *lacF* | -2.43 | 1.41 | 43.64 | 1130688 | P | Lactose transport system permease protein |
| LLNZ_RS09465 | *phoU* | -2.51 | 1.12 | 4.09 | 1875217 | P | Phosphate-specific transport system accessory protein PhoU homolog |
| LLNZ_RS03825 | *ganQ* | -6.29 | 1.15 | 6.16 | 719761 | P | Putative arabinogalactan oligomer transport system permease protein |
| LLNZ_RS05075 | *yfnB* | 3.18 | 1.12 | 4.24 | 960818 | R | Putative HAD-hydrolase |
| LLNZ_RS09555 | *pbuO* | 3.14 | 1.21 | 13.41 | 1894784 | R | Guanine/hypoxanthine permease |
| LLNZ_RS11180 | *-* | 3.01 | 1.10 | 3.15 | 2194754 | R | - |
| LLNZ_RS02345 | *-* | 3.01 | 1.12 | 4.63 | 455504 | R | - |
| LLNZ_RS01915 | *era* | 2.57 | 1.22 | 17.43 | 363169 | R | GTPase |
| LLNZ_RS12645 | *mnmE* | 2.49 | 1.18 | 10.54 | 2478313 | R | tRNA modification GTPase |
| LLNZ_RS02115 | *yycJ* | 2.49 | 1.14 | 6.22 | 406145 | R | Putative metallo-hydrolase |
| LLNZ_RS03160 | *yqjQ* | 1.84 | 1.22 | 13.61 | 593314 | R | Uncharacterized oxidoreductase |
| LLNZ_RS00460 | *ykpA* | 1.83 | 1.12 | 5.25 | 81291 | R | Uncharacterized ABC transporter ATP-binding protein |
| LLNZ_RS01210 | *yqeH* | 1.82 | 1.18 | 10.19 | 222305 | R | Uncharacterized protein |
| LLNZ_RS08990 | *cidA* | 1.62 | 1.26 | 18.77 | 1777056 | R | Holin-like protein |
| LLNZ_RS01435 | *pgfs* | -1.69 | 1.18 | 10.15 | 263199 | R | Prostaglandin F synthase |
| LLNZ_RS12715 | *-* | -1.73 | 1.20 | 13.24 | 2489820 | R | - |
| LLNZ_RS00470 | *vdlC* | -1.73 | 1.23 | 15.69 | 83066 | R | Probable short-chain type dehydrogenase/reductase |
| LLNZ_RS10290 | *pgfs1* | -1.74 | 1.18 | 9.33 | 2050053 | R | Prostaglandin F synthase |
| LLNZ_RS01285 | *-* | -1.75 | 1.15 | 6.89 | 234391 | R | - |
| LLNZ_RS00180 | *-* | -2.10 | 1.27 | 23.17 | 36121 | R | - |
| LLNZ_RS11660 | *yvdE* | -2.33 | 1.18 | 21.12 | 2286973 | R | Putative glutamine amidotransferase-like protein |
| LLNZ_RS04515 | *steap3* | -2.36 | 1.25 | 17.62 | 853855 | R | Metalloreductase |
| LLNZ_RS02205 | *cstA* | -2.47 | 1.24 | 18.43 | 422985 | T | Carbon starvation protein A homolog |
| LLNZ_RS10100 | *usp* | -3.47 | 1.10 | 2.16 | 2010508 | T | Putative universal stress protein |
| LLNZ_RS11825 | *secY* | 3.23 | 1.11 | 4.19 | 2316372 | U | Protein translocase subunit |
| LLNZ_RS02835 | *yidC* | 2.96 | 1.14 | 6.13 | 539492 | U | Membrane protein insertase |
| LLNZ_RS12060 | *comG* | -2.14 | 1.16 | 8.43 | 2349023 | U | ComG operon protein 1 |
| LLNZ_RS09280 | *lmrA* | -1.56 | 1.12 | 4.61 | 1837553 | V | Multidrug resistance ABC transporter ATP-binding and permease protein |

^a^ The Log_2_ ratio indicated log_2_foldchange(NZ*rmaH*/NZ9000).
